# In Vitro Characterization of the Published Glypican-3-Targeting Peptide TJ12P2 Reveals a Lack of Specificity and Potency

**DOI:** 10.3390/ph18111656

**Published:** 2025-11-01

**Authors:** Eva-Maria Burger, Charlice Hill, Robert Wodtke, Kristof Zarschler, Markus Laube, Cornelius K. Donat, Sandra Hauser, Klaus Kopka, Jens Pietzsch, Sven Stadlbauer

**Affiliations:** 1 Helmholtz-Zentrum Dresden-Rossendorf, Institute of Radiopharmaceutical Cancer Research, Bautzner Landstraße 400, 01328 Dresden, Germany; e.burger@hzdr.de (E.-M.B.); c.hill@hzdr.de (C.H.); r.wodtke@hzdr.de (R.W.); k.zarschler@hzdr.de (K.Z.); m.laube@hzdr.de (M.L.); donat@hzdr.de (C.K.D.); s.hauser@hzdr.de (S.H.); k.kopka@hzdr.de (K.K.); j.pietzsch@hzdr.de (J.P.); 2 Faculty of Chemistry and Food Chemistry, School of Science, TU Dresden, Mommsenstraße 4, 01069 Dresden, Germany

**Keywords:** HCC, GPC3, TJ12P2, liver cancer

## Abstract

**Background/Objectives:** The cell surface proteoglycan glypican-3 (GPC3) is reportedly overexpressed in hepatocellular carcinoma (HCC) tissues, but not in benign liver tissues, rendering this protein a potential target for radionuclide theranostic approaches. Peptides are generally a promising class of targeting molecules for the development of radioligands because they combine straightforward synthetic access with favorable pharmacokinetics. Among the published peptides with disclosed structures, one of the most promising radioligands is **[^18^F]AlF-NOTA-TJ12P2**, which has a reported comparably high binding affinity to GPC3 and a high hydrophilicity. In this study, we aimed to design novel GPC3-targeting radioligands based on the TJ12P2 peptidic scaffold. **Methods:** Peptides were synthesized on solid phase using an Fmoc protecting group strategy. For comparative investigations, the reference nanobody HN3 was expressed in *E. coli*, isolated and subsequently modified with NODA-GA or SulfoCy3. The binding of native peptides, scrambled variants and reference nanobodies to GPC3 was investigated by surface plasmon resonance (SPR) interaction analysis, and fluorescently labeled versions of peptides and nanobodies were used for fluorescence microscopy in HepG2 (GPC3+) or SK Hep1 (GPC3−) cells. The chelator-bearing peptides were radiolabeled with gallium-67 and their stability towards radiolysis and in human serum was investigated. The binding of radiolabeled peptides and nanobodies to HepG2 cells was assessed in real-time ligand binding experiments. **Results:** The synthesized native peptides did not exhibit binding towards GPC3 in SPR interaction analyses, and the observed response was comparable to that of the scrambled variants at equal concentrations. Additionally, no binding to or uptake of the fluorescent constructs into cells was observed with fluorescence microscopy regardless of cellular GPC3 expression level. In real-time radioligand binding experiments, very fast association and dissociation of the gallium-67 labeled peptides to GPC3 positive HepG2 cells was observed, suggesting either extremely fast binding kinetics or unspecific binding of the peptides. **Conclusions:** Taken together, these findings suggest that the peptide TJ12P2 lacks specific binding to GPC3 in vitro and might not serve as a basis for the development of radioligands targeting GPC3.

## 1. Introduction

As of 2022, liver cancer is the sixth-most-common type of cancer worldwide, and the third-most-common cause of cancer-related deaths [1]. Approximately 80% of those cases are attributed to HCC [2,3]. Since it is often diagnosed at a late stage, HCC is associated with high mortality. Only 20–30% of late-stage patients survive for longer than five years after diagnosis. In contrast, the five-year survival rates for patients diagnosed at an early stage of disease are considerably higher (60–70%), owing to the variety of applicable treatment options such as liver resection and transplantation [4,5]. Appropriate diagnostic options can therefore help with early detection and subsequent treatment. Non-invasive molecular imaging, such as positron emission tomography (PET), is well suited for this task, as exemplified by the PET tracer [^18^F]FDG for the highly sensitive monitoring of glucose utilization in a variety of cancer entities [6]. However, the performance of [^18^F]FDG in the context of HCC is subpar due to variable tumor uptake and retention, with reported sensitivities of 30–70% [7,8]. To date, no targeted PET tracer for HCC is in routine clinical use. Therefore, the development of HCC-specific tracers could significantly improve diagnosis and thus potentially improve clinical outcomes for patients.

GPC3 has been discussed as a potential target for the development of a HCC-specific radiotracer. The upregulation of this cell surface-bound proteoglycan has been reported in HCC, but not in surrounding benign liver tissue [9,10,11]. The feasibility of GPC3-targeted radiotracers has been demonstrated using a variety of targeting vectors. Radiolabeled antibodies, such as iodine-124 codrituzumab, [^89^Zr]Zr-Df-BAY3547926, [^89^Zr]Zr-Df-αGPC3 and [^89^Zr]Zr-Df-H3K3 have been successfully employed to visualize GPC3-positive lesions both in animal models and humans [12,13,14,15]. Tumor-to-liver ratios higher than 30 were reported in some cases, both in animal models and human patients [12,16]. However, the highest contrasts were typically observed after seven days, owing to the rather slow kinetics of antibodies, hindering application in clinical practice [12,13,14]. Efforts to overcome this problem yielded the nanobodies [^89^Zr]Zr-Df-ssHN3 and [^68^Ga]Ga-NOTA-G2/[^18^F]F-G2, which display significantly faster tumor uptake and shorter circulation times. However, the observed tumor-to-liver contrast was substantially lower compared to full-length antibodies, with tumor-to-liver ratios of only 1–3.5 in mice [17,18]. Another frequently employed approach for GPC3-targeted radiotracers with faster pharmacokinetics are GPC3-binding peptides, generated via phage display screening. Among the peptides described in the literature so far, most show moderate binding affinity towards GPC3 in vitro, with dissociation constants (*K*_D_) in the range of 45–750 nM [19,20,21,22,23]. Some of the described peptides also suffer from a high lipophilicity, leading to increased uptake in the liver, which is not directly related to the target expression but rather based on its role as a metabolizing organ [19,20,21,23]. Hence, tumor-to-liver contrasts are moderate, with reported values between 0.9 and 3 [19,20,21,22]. Among the published GPC3-binding peptides with disclosed structures, 12mer **TJ12P2** exhibits the most promising properties. The peptide has been reported by *Qin* et al. in 2020 as a highly hydrophilic peptide from phage display which shows a binding affinity of 158 nM to GPC3. Furthermore, the radiolabeled analog **[^18^F]AlF-NOTA-TJ12P2** was found to display specific accumulation in GPC3-expressing tumors as well as tumor-to-liver contrasts of up to 2.85, rendering this peptide promising for further clinical translation [22]. However, doubts about the in vitro specificity and potency of other linear GPC3-binding peptides like **TJ12P1** and **L5** have been raised based on biolayer interferometry data and GPC3-dependent cellular uptake experiments [24,25]. In this context and in connection with this work, we have become aware of contradictory information in the publication by *Qin* and coworkers which specifically concerns the reported amino acid in position 11. While all written sequences contained a lysine in position 11, all chemical structures were shown with a glutamine in position 11. Of note is also the lack of data on the serum stability of the radioligand provided by the authors.

Within our efforts to develop GPC3-targeting radiotracers, we aimed to develop ^68^Ga- or ^64^Cu-labeled derivatives of **TJ12P2** with improved tumor-to-liver contrast by identifying structure–activity relationships and investigating the human serum stability of the peptides. In the beginning and to confirm the reported binding of the peptide **TJ12P2** to the protein, we decided to synthesize the corresponding native peptides **TJ12P2(Q^11^/K^11^)** and scrambled variants to investigate the influence of amino acid substitution in position 11. As a starting point for our own tracer developments, we chose to synthesize variants with an N-terminally attached fluorophore moiety (**SulfoCy3-TJ12P2(Q^11^/K^11^)**) as well as derivatives with an N-terminally and C-terminally conjugated NODA-GA chelator (**NODA-GA-TJ12P2(Q^11^/K^11^)** and **TJ12P2(Q^11^/K^11^)-X_3_K(NODA-GA)-CONH_2_**). As a reference for comparative in vitro characterization, we produced NODA-GA and SulfoCy3 derivatives of the literature-known nanobody HN3 which binds to the N- and C-terminal domains of GPC3 simultaneously and hence the correctly folded core protein [17,26]. The binding of the native peptides and the nanobody to GPC3 was assessed by SPR interaction analysis. Furthermore, the fluorophore-labeled conjugates were used for the imaging of human HCC cells with high (HepG2) and low (SK-Hep1) GPC3 expression. Additionally, the chelator-bearing constructs were radiolabeled with gallium-67, and serum stability as well as real-time binding to living HepG2 cells were investigated. No further developments were made due to the absence of specific binding to GPC3 in the performed investigations as reported in detail herein.

## 2. Results

### 2.1. Synthesis and Characterization

**Figure 1:**
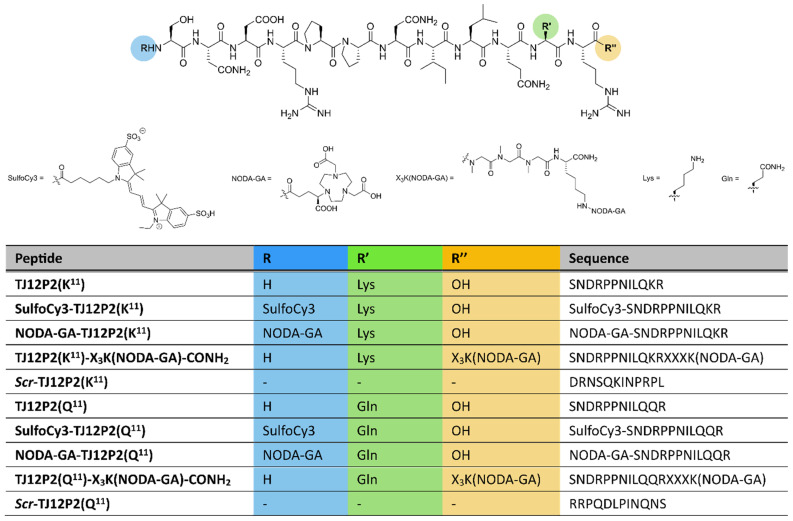
Chemical structure of synthesized **TJ12P2** conjugates as well as peptide sequences for **TJ12P2** conjugates and scrambled variants. Sarcosine is denoted as X. Structure of ***scr*-TJ12P2(Q^11^/K^11^)** is only shown as amino acid sequence in the table.

The target peptides **TJ12P2(Q^11^/K^11^)** and ***scr*-TJ12P2(Q^11^/K^11^)** were successfully synthesized by standard solid-phase peptide synthesis using N^α^-Fmoc-protected amino acids with orthogonal protecting groups for side-chain functionalities. **NODA-GA-TJ12P2(Q^11^/K^11^)** and **SulfoCy3-TJ12P2(Q^11^/K^11^)** were synthesized by the coupling of (*t*Bu)_3_NODA-GA or SulfoCy3 to the N-terminus of **TJ12P2(Q^11^/K^11^)** on resin. For the C-terminal chelator-bearing peptide, **Fmoc-TJ12P2(Q^11^/K^11^)-X_3_K(Alloc)** was synthesized on resin. Removal of the Alloc group, followed by amide coupling of (*t*Bu)_3_NODA-GA to the free lysine and subsequent deprotection and cleavage gave **TJ12P2(Q^11^/K^11^)-X_3_K(NODA-GA)-CONH_2_**. The synthesized peptide constructs are depicted in Figure 1, and synthetic schemes are summarized in Figures S1–S4.

All peptides were purified using semipreparative RP-HPLC, and their purity was assessed by an analytical RP-HPLC. The determined purity of all peptides was found to be higher than 95%. The HRMS of the purified peptides was found to be in accordance with the calculated values (Table 1).

A version of the literature-known nanobody HN3 with a C-terminal LPETG sortase motif and TwinStrep tag, **HN3-sortag-(Strep)_2_**, was obtained by cytoplasmic expression in *Escherichia coli* and subsequent purification was performed via affinity chromatography as described by *Singh* and coworkers [26,27]. An azidolysine moiety was introduced site-specifically at the sortase motif of HN3 using transpeptidase Sortase A Δ59. Then, BCN-NODA-GA or DBCO-SulfoCy3 was introduced by strain-promoted alkyne–azide cycloaddition to the azide group. The synthetic procedure is depicted in Figure S25.

### 2.2. SPR Interaction Analysis Experiments

**Figure 2:**
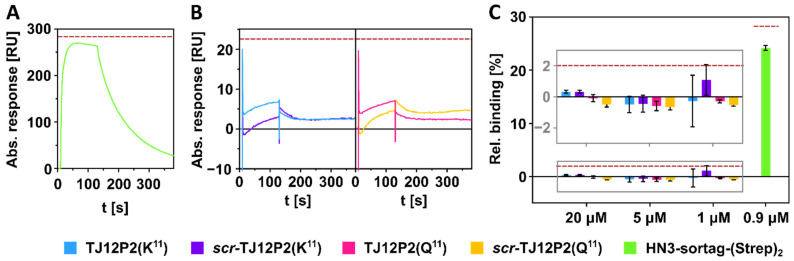
Double-referenced sensorgrams of the nanobody **HN3-sortag-(Strep)_2_** (900 nM, (**A**)) and peptides (20 μM, (**B**)). Relative binding of the peptides at different concentrations and control nanobody at 900 nM (**C**). Maximum observable signal at observed capture level of GPC3 (ca. 1200 RU) is indicated by a dark red dashed line.

In order to check the reported binding of the native peptides to GPC3 and evaluate the potential influence of the amino acid in position 11 on the binding behavior, GPC3 interaction was assessed by SPR interaction analysis using His-tagged recombinant human GPC3 as ligand. For the initial screening of potential binders and to determine a suitable concentration range for further analyses, GPC3 was captured on an anti-His-antibody functionalized CM5 chip and the binding response of analytes at different concentration was analyzed after 120 s association time. Furthermore, blank runs were performed to obtain double-referenced sensorgrams which help to evaluate the binding response. The binding of the nanobody **HN3-sortag-(Strep)_2_** to GPC3 was determined as a reference. **HN3-sortag-(Strep)_2_** bound to GPC3 showed a signal response expected for 1:1 binding at a concentration of 900 nM leading to immediate saturation and hence served as suitable positive control (Figure 2). Of note is that further single-cycle kinetic experiments were performed using **HN3-sortag-(Strep)_2_** and **NODA-GA-HN3** that revealed dissociation constants of 29.6 nM and 8.50 nM, respectively, for binding to GPC3 (Figures S26 and S27). In contrast, native peptides **TJ12P2(Q^11^/K^11^)** did not show any binding to GPC3 independently of the applied concentration (Figure 2C), which was derived from analyzing the observed binding signal in relation to the potential binding response as expected from 1:1 binding based on captured GPC3. Furthermore, no significant difference in signal intensity was observed between the native peptides **TJ12P2(Q^11^/K^11^)** and their respective scrambled variants ***scr*-TJ12P2(Q^11^/K^11^)** (Figure 2B). Due to the absence of binding response or specificity, no further SPR analyses were performed to determine dissociation constants.

### 2.3. Fluorescence Microscopy

**Figure 3:**
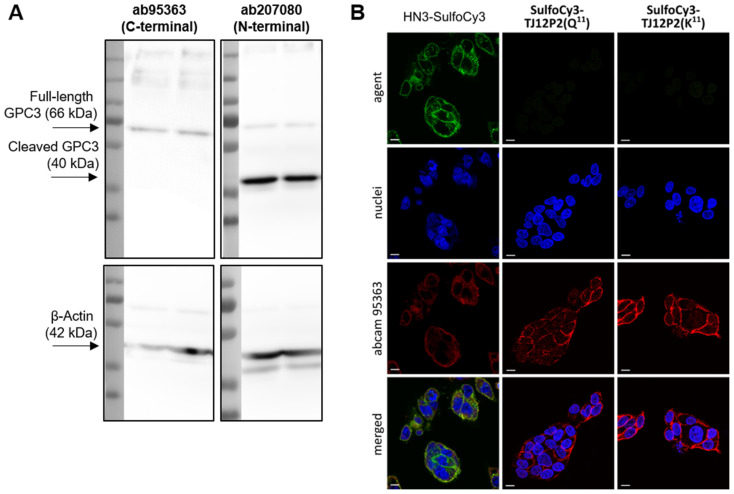
(**A**) Western blot images generated using primary antibodies binding C-terminal (ab95363) or N-terminal (ab207080) epitopes of GPC3 in two different HepG2 lysates. β-Actin was used as a loading control. Uncropped/whole Western blot images are provided in Figures S29 and S30. (**B**) Fluorescence microscopy images (scale bars: 10 μm) of GPC3-positive HepG2 cells stained using **SulfoCy3-HN3**, **SulfoCy3-TJ12P2(Q^11^)** and **SulfoCy3-TJ12P2(K^11^)** (1 μM, green) after 2 h of incubation. Nuclei, stained with DRAQ5, are depicted in blue, and the control stain of GPC3 using ab95363 is depicted in red.

The fluorophore-labeled peptides and nanobody conjugates were incubated with human GPC3-positive HCC cells (HepG2, Figure 3). Expression of the full-length GPC3 protein in the cell line was orthogonally confirmed via Western blot (Figure 3). A commercially available antibody (abcam95363) was employed as an additional control and to demonstrate target expression via co-localization. On HepG2 cells, an overlap of the fluorescence signals of the reference antibody and the SulfoCy3-labeled nanobody construct was observed. In contrast, GPC3-negative SK-Hep1 cells exhibited only negligible fluorescence signals after incubation with both compounds (Figure S28). Importantly, no fluorescence signal was observed for both **SulfoCy3-TJ12P2(Q^11^)** and **SulfoCy3-TJ12P2(K^11^)**, either in target positive HepG2 or negative SK-Hep1 cells.

### 2.4. Radiolabeling and Characterization

All chelator-bearing peptide constructs were successfully radiolabeled with [^67^Ga]GaCl_3_ in ammonium acetate-buffered solution (pH 4.3) at 60 °C for 30 min. Each conjugate was labeled with a radiochemical purity of more than 98%, and apparent molar activities were between 3.30 and 5.90 GBq/μmol (non-labeled peptide not separated after radiolabeling). The partition coefficients between octanol and PBS (pH 7.4) of all radiolabeled peptides were lower than −4.50, indicating a high polarity of the peptides (Table 2). Furthermore, all peptides were stable towards radiolysis in both labeling buffer and PBS for 4 h and 24 h, respectively. Stability investigations in human serum revealed that the C-terminally labeled constructs were substantially more stable than the N-terminally labeled constructs. After 24 h in human serum, 89% and 95% of **TJ12P2(K^11^)-X_3_K([^67^Ga]Ga-NODA-GA)-CONH_2_** and **TJ12P2(Q^11^)-X_3_K([^67^Ga]Ga-NODA-GA)-CONH_2_** were still intact. **[^67^Ga]Ga-NODA-GA-TJ12P2(Q^11^)** is slightly less stable, with only 72% of radioligand intact after 24 h. **[^67^Ga]Ga-NODA-GA-TJ12P2(K^11^)** degrades rapidly in human serum, with only 9.3% of the radioligand still intact after 1 h (Figure S31). The formation of a single metabolite was observed, and a serum half-life of 17.4 min was calculated for **[^67^Ga]Ga-NODA-GA-TJ12P2(K^11^)**. LC-MS analysis of the corresponding **SulfoCy3-TJ12P2(K^11^)** after incubation in human serum for 24 h revealed that the C-terminal arginine residue was cleaved.

The reference nanobody **NODA-GA-HN3** was radiolabeled using [^64^Cu]CuCl_2_ in ammonium acetate buffer (pH 6.0) at 37 °C for 30 min. The desired radiolabeled nanobody was obtained in an apparent molar activity of 30 MBq/nmol. While gallium-67 was chosen for peptide labeling as a direct surrogate for the widely clinically used gallium-68, **NODA-GA-HN3** was labeled with copper-64 due to the milder labeling conditions required.

### 2.5. Real-Time Radioligand Binding

**Figure 4:**
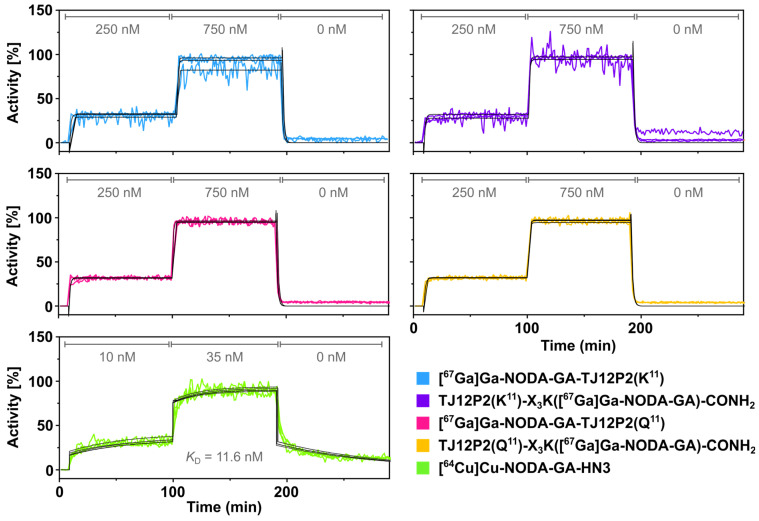
Real-time ligand binding of radiolabeled peptides and **[^64^Cu]Cu-NODA-GA-HN3** to live HepG2 cells. Experiments were performed in triplicates for radiolabeled peptides and as quintuplicate for radiolabeled HN3. Data shows signal (activity, decay corrected) in the target area (HepG2 cells), with the background area (no cells) subtracted and then normalized to account for differences in cell density and molar activity.

Binding of the radiolabeled chelator-substituted **TJ12P2(Q^11^/K^11^)** and nanobody to human hepatoma cells with high GPC3 expression was investigated via real-time radioligand binding. Here, vital cells (target) and a control (background, no cells) in a Petri dish were alternately incubated with the radioligand. Over time, stable binding appeared as a signal increase, from which the background was subtracted. Pilot experiments indicated no apparent binding (flat signal) of the peptides at low concentrations (<50 nM). Hence, we employed higher concentrations of 250 nM and 750 nM. At these concentrations, all radiolabeled peptides exhibited the same pattern: in both association phases, an almost immediate initial signal increase occurred, followed by a stable plateau. Similarly, the dissociation phase was characterized by a sudden signal decrease, again followed by a stable plateau. This pattern was observed for both the target and background area, albeit less prominent in the latter (Figure 4). Raw data (CPS), provided in the Supplementary Materials (Figure S32), for all four compounds illustrates the described pattern.

The resulting signal (target-background) exhibited no discernible curvature (signal increase/decrease over time) in the association phase. Additionally, only very little and apparently very fast dissociation was observed. Sufficient curvature is required for reliable data evaluation and calculation of kinetic constants. Hence, no reliable binding data was derived from measurements up to 750 nM.

In contrast, the reference nanobody **[^64^Cu]Cu-NODA-GA-HN3** yielded results clearly consistent with binding. During the association phase, a gradual increase in radioactivity over time was found in the target area (HepG2 cells), which was absent in the background. This increased further at a higher concentration of **[^64^Cu]Cu-NODA-GA-HN3**. When the medium containing the compound was replaced, a clear decrease in signal over time was observed, indicating dissociation of the bound tracer. Data was fitted using a 1:1 model accounting for bulk effect which yielded a *K*_D_ of 11.6 nM.

## 3. Discussion

During the past decade, GPC3 has emerged as a promising target for HCC imaging, owing to its high and low abundance in malignant and normal liver cells, respectively. A range of imaging agents based on linear peptides have been developed, but most of them suffer from certain drawbacks, such as a high lipophilicity, a low binding affinity or conflicting reports about their suitability for targeting GPC3 [19,20,21,22]. Among the reported GPC3 binders with disclosed structures so far, the peptide **TJ12P2** appeared the most promising due to its hydrophilic nature and reported GPC3-binding affinity of 158 nM [22]. This prompted us to develop novel radiolabeled analogs of **TJ12P2** for the imaging of tumor-associated GPC3.

The native peptide, a scrambled variant, a fluorescent analog and two constructs with N- and C-terminal chelator substitution were synthesized. Since the peptide sequence for **TJ12P2** is ambiguously reported, with either a lysine or a glutamine in position 11, the listed derivatives were synthesized for both variants. The binding of the native peptides and scrambled variants to GPC3 was assessed via SPR interaction analysis. No binding was observed for the native peptides **TJ12P2(Q^11^)** and **TJ12P2(K^11^)**, contrary to the report of *Qin* and coworkers. The observed responses for both peptides were similar to responses obtained for the respective scrambled variants. As a positive control, we investigated the binding of the nanobody variants **HN3-sortag-(Strep)_2_** and **NODA-GA-HN3 [26]**. Both versions showed the expected concentration-dependent binding, and the determined dissociation constants were 29.6 nM and 8.50 nM, respectively. We expect that the slightly different binding affinities are a result of different N-terminal substitutions between the two analogs. The determined values are in accordance with the value reported for [^89^Zr]Zr-Df-ssHN3, which has a dissociation constant of 10 nM in cell-free saturation experiments [17]. Since this nanobody reportedly identifies a conformational epitope including both N- and C-terminal domains of GPC3, this proves that the core protein of GPC3 attached to the chip is complete and functional [26]. We initially speculated that the observed lack of binding could be a result of altered GPC3 processing in the recombinant expression system. To rule out this possibility, additional microscopy experiments were conducted using fluorescently labeled conjugates of the peptides in human liver cancer cells.

GPC3-positive (HepG2) and negative (Sk-Hep1) cells were incubated with SulfoCy3-labeled variants of the peptides (**SulfoCy3-TJ12P2(K^11^/Q^11^)**) as well as a commercial control antibody (ab95363). No fluorescent signal was observed for the peptides on either cell line, indicating neither a binding nor an uptake of the fluorophore-labeled probes. In contrast, the expected co-localization of a fluorescent signal of the antibody and a fluorescent nanobody conjugate (**SulfoCy3-HN3**) was observed in HepG2 cells. Negligible fluorescent signals were observed in either channel in Sk-Hep1 cells, confirming expected GPC3 expression patterns and selectivity. To ensure that the lack of surface binding of the peptides was not caused by extensive washing or subsequent fixation, additional control experiments were conducted using radiolabeled peptides and nanobody conjugates in living HepG2 cells.

A real-time live-cell radioligand assay further supported absence of specific binding for the radiolabeled peptides. Here, only a very high and fast sudden increase in signal was observed in response to changes in ligand concentration. No dissociation was observable, again, just a sudden drop in signal. Furthermore, it is apparent from the raw data that signal shape over time was virtually identical between the background and target areas. Combined with all other data, this primarily indicates a lack of specific binding, with signal changes most likely to occur due to fast, non-specific binding processes. This is further substantiated by the raw data. Considering that HepG2 cells provide sufficient target expression, having a nearly identical signal pattern in thr presence and absence of these cells strongly precludes specific binding.

However, other reasons cannot be ruled out completely. Such a signal pattern could theoretically be attributed to a considerably higher *K*_D_, specifically as a result of both extremely fast association and dissociation rate constants. While it would be possible to observe this through low concentrations and fast intervals, another technique would be to further increase the concentration. If saturation could be achieved, this would indicate at least some specific binding. Again, considering all other data and specifically SPR interaction analyses, we conclude that the binding of the peptides is dominated by the non-specific binding to cells and a lack of specific binding to GPC3, rather than fast but non-potent binding to GPC3. In contrast, **[^64^Cu]Cu-NODA-GA-HN3** bound to HepG2 cells with a binding affinity of 11.6 nM, very similar to the value determined for **NODA-GA-HN3** via SPR and thus in good agreement with a comparable HN3 construct from the literature [17].

Stability studies of the radiolabeled peptides in buffer showed that the conjugates are stable towards radiolysis as described in the literature. In human serum, the peptide **[^67^Ga]Ga-NODA-GA-TJ12P2(K^11^)** was rapidly cleaved, with a half-life of only 17.8 min while all other constructs were reasonably stable over 24 h. Further investigations revealed that the C-terminal arginine is cleaved, suggesting the involvement of exopeptidases, possibly carboxypeptidases [28,29]. This could likely be avoided by exchanging the C-terminal carboxy moiety for an amide.

Taken together, our results suggest that the reported peptide **TJ12P2** does not exhibit specific binding to GPC3, regardless of which amino acid is situated in position 11. Of note is that for the GPC3-targeting peptide **TJ12P1** which was created by the same group [20], multiple recent in vitro studies could also not confirm binding to GPC3 [24,25]. However, we still expect GPC3-targeting peptides, in general, to be promising for radiotracer development. For example, it was recently shown that GPC3-targeting peptides show promise as theranostic agents for HCC. SPECT imaging of **[^177^Lu]Lu-DOTA-RAYZ-8009** showed promising tumor accumulation in vivo, and therapeutic efficiency was demonstrated in animals [30]. Furthermore, the peptide **[^68^Ga]Ga-DOTA-RAYZ-8009** was shown to selectively bind to GPC3-positive HCC lesions in humans [31,32]. The structure of **RAYZ-8009** has not been disclosed to date, which impedes independent research efforts. However, the reported results demonstrate that not only HCC imaging but also therapy using GPC3-targeting peptidic radioligands are highly promising and have the potential to reshape the clinical landscape in the future [30,31,32].

## 4. Materials and Methods

### 4.1. Peptide Synthesis and Characterization

All peptides reported in this manuscript were synthesized using an Fmoc-base protecting strategy on solid phase, purified by preparative HPLC and characterized by HRMS. Purity was determined by analytical HPLC. Detailed synthetic schemes, analytical HPLC chromatograms and HRMS spectra can be found in Supplementary Materials Section S1.

### 4.2. Nanobody Synthesis and Isolation

A tagged version of the literature nanobody HN3 **(HN3-sortag-(Strep)_2_)** was obtained by cytoplasmic expression in *Escherichia coli* and subsequent purification via affinity chromatography as described by *Singh* and coworkers [27]. The introduction of an azidolysine moiety was carried out site-specifically at the C-terminal LPETG sortase motif using Sortase A Δ59. Clicking of DBCO-SulfoCy3 or BCN-NODA-GA by strain-promoted alkyne–azide cycloaddition to the azide group yielded the constructs **SulfoCy3-HN3** and **NODA-GA-HN3**. A schematic depiction and detailed descriptions of the procedures can be found in Supplementary Materials Section S2.

### 4.3. SPR Interaction Analysis

Binding analysis of the peptides and nanobodies to GPC3 via SPR interaction analysis was carried out on a Biacore T200 (GE Healthcare, Chicago, IL, USA) with CM5 sensor chips (29149603, Cytiva, Marlborough, MA, USA) and HBS-P+ running buffer (BR100827, Cytiva, Marlborough, MA, USA). Measurements were conducted at 25 °C using a flow rate of 30 μL/min unless specified otherwise, and datapoints were collected in 0.1 s intervals. The normalization of all flow cells (FCs) was carried out, and two consecutive FC were functionalized using the His capture kit (29234602, Cytiva, Marlborough, MA, USA) and an amine coupling kit (BR100050, Cytiva, Marlborough, MA, USA). Applying the procedures according to the manufacturer’s protocol, and the immobilization of the anti-His antibody yielded signals of 2607–3032 RU and 2645–3113 RU for the reference and active flow cell on the chip, respectively.

The binding of the peptides and HN3 as positive control was analyzed in a multi-cycle kinetic starting by capturing GPC3 (5 μg/mL in HBS-P+, 120 s; 10 μL/min) on the active flow cell. After a stabilization period of 280 s, **HN3-sortag-(Strep)_2_** (900 nM) or different concentrations (20 μM, 5 μM, 1 μM) of the respective peptides were injected over a period of 120 s followed by a dissociation phase of 300 s. The chip was subsequently regenerated as described above. Blank runs using only buffer (HBS-P+) were conducted to enable double referencing.

Data were evaluated using the Biacore Evaluation software 3.0. Reference subtraction (FC(active)−FC(reference)) and blank correction were carried out for each sensorgram. For nanobody dissociation constant determination, data were analyzed according to a 1:1 stochiometric binding model. For the binding analysis of the peptides and **HN3-sortag-(Strep)_2_**, relative binding was calculated by measuring the signal at t_binding_ (at end of association) of the sample with GPC3 capture (RU_analyt_), buffer with GPC3 capture (RU_blank_) and buffer without GPC3 capture (RU_baseline_). Relative response was calculated as follows: Relative Response = (RU_analyt_ − RU_blank_)/(RU_blank_ − RU_baseline_). Expected relative maximum response (relative RUmax) was calculated by dividing the molar mass of the analyte with the molar mass of GPC3. The expected absolute maximum response (RUmax) in double-referenced chromatograms was calculated by multiplying the relative RUmax with the signal of captured GPC3 (RU_blank_ − RU_baseline_).

Procedures for the single cycle-kinetics of **HN3-sortag-(Strep)_2_** and **NODA-GA-HN3** and corresponding calculated dissociation constants are reported in Supplementary Materials Section S3.

### 4.4. Cell Culture

HepG2 and Sk-Hep1 cells were obtained from ATCC and cultured in Dulbecco’s Modified Eagle Medium (31966, Gibco, Waltham, MA, USA) or Eagle’s Minimum Essential Medium with Glutamine (30-2003, ATCC, Manassas, VA, USA), respectively, supplemented with 10% fetal calf serum (FBS, F7524, Sigma-Aldrich, St. Louis, MO, USA), penicillin (100 U/mL) and streptomycin (100 µg/mL; Pen Strep, 15140, Gibco, Waltham, MA, USA) at 37 °C and 5% CO_2_ in a humidified incubator. GPC3 expression was tested by Western blotting.

### 4.5. Western Blotting

Cells were lysed in a RIPA buffer (150 mM NaCl, 50 mM Tris pH 8.0, 1% NP40, 0.5% *w*/*v* SDS, 7 μg/mL leupeptin, 1 mM PMSF, 1 mM Na_3_VO_4_, 1 mM DTT, and 7 mM NaF) and the protein concentration was determined by bicinchoninic acid assay. Lysates were diluted to 1 mg/mL and denatured for 10 min at 95 °C by the addition of 5x protein sample buffer (312.5 mM Tris pH 6.8, 40% glycerol, 10% SDS, 5% β-mercaptoethanol, and 0.1% bromophenol blue). SDS-PAGE was run using a 10% separation gel and proteins were transferred to a PVDF membrane by Western blotting. Blots were blocked with 5% skimmed milk in Tris-buffered saline containing 0.05% Tween and incubated with primary antibodies (anti-GPC3, ab95363, Abcam, Cambridge, UK, binds C-terminal, 1:1000; anti-GPC3, ab207080, Abcam, Cambridge, UK, binds N-terminal, 1:1000; and anti-β-Actin, A5316, Sigma-Aldrich, St. Louis, MO, USA, 1:5000) diluted in blocking solution overnight. After washing, blots were incubated with secondary antibodies (anti-rabbit POD, A0545, Sigma-Aldrich, St. Louis, MO, USA, 1:5000 and anti-mouse POD, A9044, Sigma-Aldrich, St. Louis, MO, USA, 1:10,000) for 1 h and washed again before the application of the chemiluminescent substrates SuperSignal West Pico and Femto (34580 and 34096, Thermo Fisher, Waltham, MA, USA) for 1 min and the detection of chemiluminescence using a Biostep CELVIN^®^ S 420 FL Imager (Biostep GmbH, Burkhardtsdorf, Germany).

### 4.6. Fluorescence Microscopy

In 8-well chamber slides (ibidi, Gräfelfing, Germany), 1 × 10^6^ HepG2 cells and 2.5 × 10^5^ Sk-Hep1 cells per well were seeded 24 h before and cultured to a confluence of approximately 80%. At first, cells were incubated with a concentration of 1 µM fluorescently labeled peptide or **SulfoCy3-HN3** in culture medium for 2 h at 37 °C. A negative control was treated with the same amount of cell medium only. In order to confirm GPC3 expression by the HCC cells and to investigate the possible co-localization of the fluorescently labeled peptides or nanobody conjugates with cellular GPC3, the commercially available reference antibody ab95363 (Abcam, Cambridge, UK) was used with a concentration of 1 µM in culture medium and was also incubated for 2 h at 37 °C. After the incubation time, the medium was carefully removed, and cells were washed with 0.5% bovine serum albumin in PBS for 5 min three times each. After that, cells were fixed using 4% paraformaldehyde and 2.5% sucrose in PBS for 20 min at ambient temperature. Additionally, cell nuclei were stained with DRAQ5 (1:1000 in PBS of 5 µM stock) for 10 min at ambient temperature. Afterwards, the staining solution was removed and cells were washed twice with PBS for 5 min. Fluorescence microscopic imaging was performed using a Fluoview 1000 confocal laser scanning microscope (Evident, Hamburg, Germany).

### 4.7. Radiochemistry

^67^Ga was produced via the ^68^Zn(p,2n)^67^Ga nuclear reaction using a TR-Flex (Advanced Cyclotron Systems, Inc., Richmond, BC, Canada) cyclotron [33]. For ^67^Ga labeling, 10 μL of [^67^Ga]GaCl_3_ solution (2.25–4.36 GBq/mL) in aqueous HCl (50 mM) was brought to pH 4.3 using 98 μL aqueous NH_4_OAc (0.5 M, pH 4.5). To the buffered solution 2–5 nmol peptide in DMSO (final concentration of 30–50 μM) was added, and after thorough mixing with a pipet, the mixture was incubated at 60 °C for 30 min. The completion of the reaction was monitored by TLC using silica-coated glass microfiber chromatography paper (iTLC-SG, Agilent, Santa Clara, CA, USA) in citrate buffer (0.1 M, pH = 4). Free [^67^Ga]Ga (as citrate complex) migrates with the solvent front (R_f_ = 1), while the radiolabeled peptides remain at the origin (R_f_ = 0). Since labeling yields were greater than 95%, the obtained solutions were used directly in subsequent experiments. Radiolabeled peptides were obtained in apparent molar activities (non-labeled peptide was not removed after labeling) in the range of 4.5–16.7 GBq/μmol at the end of synthesis.

[^64^Cu]CuCl_2_ was produced via the ^64^Ni(p,n)^64^Cu nuclear reaction using a TR-Flex (Advanced Cyclotron Systems, Inc., Richmond, BC, Canada) cyclotron as reported previously [33]. ^64^Cu-labeling was performed using ~30 MBq of [^64^Cu]CuCl_2_ per nmol of the NODA-GA-functionalized nanobody, and the labeling reactions were set up in 0.2 M ammonium acetate buffer (pH 6) and incubated at 37 °C for 30 min with gentle shaking. The extent of radiometal complexation was assessed by radio-TLC using silica gel TLC plates as the stationary phase and 50 mM EDTA solution (pH 5) as the mobile phase. Free [^64^Cu]Cu (as EDTA complex) migrates with the solvent front (R_f_ = 1), whereas the radiolabeled nanobodies remain at the origin (R_f_ = 0).

### 4.8. LogD_7.4_ Determination

The obtained solutions from ^67^Ga labeling were diluted with water in a 1:1 ratio and brought to pH 7 using aqueous NaOH (1 M). For Log*D*_7.4_ determination, 10 μL of neutralized labeling solution was diluted with 290 μL 1× PBS (pH 7.4) and 300 μL 1-Octanol (both pre-saturated by each other). The mixture was vortexed vigorously for 30 s and subsequently centrifuged for 5 min at 16,100 rcf. Defined volumes of each layer (10 μL for PBS, 200 μL for Octanol) were taken and analyzed for their radioactivity (ISOMED 100, Nuvia, Frankfurt, Germany). The partition coefficient Log*D*_7.4_ was determined from the decadic logarithm of the fraction of the radioactivity content of the aqueous and octanol layers.

### 4.9. Stability Studies

For human serum stability investigation, 60 μL of the ^67^Ga labeling mixture was mixed with 540 μL human serum and incubated at 37 °C. Aliquots of 25 μL were taken after 1, 2, 4 and 24 h. The aliquots were mixed with 75 μL of a mixture called “Supersol” (20% *v*/*v* EtOH, 0.5% Triton X-100, 5 mM EDTA, 0.5 mM *o*-phenantroline, and 0.1% saponine in water) and put on ice for 5 min. Subsequently, the mixtures were centrifuged at 16,100 rcf, 4 °C for 5 min. The supernatant was analyzed by analytical radio-HPLC. Serum half-lives were determined by non-linear regression according to one-phase decay. To determine the stability in labeling buffer, the labeling mixture was left at ambient temperature for 4 h. Subsequently, an aliquot of 5 μL was mixed with 45 μL of Supersol and analyzed with analytical radio-HPLC.

Analytical radio-HPLC was performed on a modular unit consisting of an operating unit (smartline manager 500, Knauer, Berlin, Germany), a pump unit (smartline pump 1000, Knauer, Berlin, Germany), a photo diode array detector (smartline PDA 2800, Knauer, Berlin, Germany) and a γ-detector (Ramona Star, Raytest, Lüttich, Belgium). The system was operated by the software Chromgate 2.8. Chromatography was conducted on a C18 column (Eurospher 100-5, 250 × 4 mm, Knauer, Berlin, Germany) at a flow rate of 1 mL/min. The following method was employed for analysis:

95:5 3 min; 95:5 → 45:55 20 min; 45:55 → 5:95 1 min; 5:95 5 min; 5:95 → 95:5 1 min; and 95:5 7 min.

### 4.10. Real-Time Radioligand Binding Using the Ligand Tracer System

HepG2 cells were cultured under the previously mentioned conditions. Prior to the experiments, cells were washed, trypsinized (0.05% Trypsin-EDTA, Gibco, Waltham, MA, USA), taken up in medium and the number of cells was determined using a CASY1 cell counter (Model TT, Schaerfe System, Reutlingen, Germany). For real-time radioligand binding, 0.25–0.6 × 10^5^ cells/mL (3 mL total volume) were seeded into one side of a Petri dish (Nunclon, # 150350, ThermoFisher, Waltham, MA, USA) ~24–48 h before experiments. Dishes were cultured on an inclined base, so that cells were located in one quadrant.

For the determination of binding kinetics (association rate constant *k*_a_ and dissociation rate constant *k*_d_), real-time assay systems (LigandTracer Yellow/White; Ridgeview Instruments AB, Uppsala, Sweden) were utilized. These systems rotate the 100 mm Petri dish on an inclined base. Cells in medium (3–4 mL total volume) are located on the lower quadrant, while the signal (radioactivity, counts per second, and decay corrected) is detected on the opposite, elevated side. The dish rotates in set intervals (typically 30 s) enabling the continuous measurement of the two alternating parts of the dish for bound radioligand (cells) against background (no cells). All experiments were conducted at room temperature, using CO_2_-independent medium (Gibco, Waltham, MA, USA). Following initial pilot experiments, *k*_a_ was determined from binding to HepG2 cells via two distinct concentrations (250/750 nM for radiolabeled peptides and 10/35 nM for the radiolabeled nanobody, additive protocol, 90 min each). Followed by replacement with fresh medium, *k*_d_ was observed for at least 90 min. This approach has been shown to yield reliable kinetic measurements in a 1:1 interaction setting [34]. No cell detachment was observed by the end of the experiments.

Binding data was evaluated using the TraceDrawer (1.9.2, Ridgeview Instruments AB, Uppsala, Sweden). Traces were imported, any spikes (>100% sudden increases in CPS over previous datapoints) were removed and each trace was normalized to its own baseline (=0%) and highest value (=100%, typically at the very end of the second association phase). This allows for the comparison of data across experiments and global fitting. Typically, 3–5 individual runs per ligand were performed. Data showing suitable binding (as indicated by curvature) was fitted in TraceDrawer using a 1:1 interaction model, with/without accounting for bulk effect.

## Figures and Tables

**Table 1:** Chemical formula, calculated m/z, m/z found in HRMS and chemical purity at 220 nm for all synthesized peptides.

Peptide	Chem. Formula	*m*/*z* calcd.	*m*/*z* found	Purity [%]
**TJ12P2(K^11^)**	C_60_H_104_N_22_O_19_	719.3997 [M+2H]^2+^	719.3994	≥99.8
**SulfoCy3-TJ12P2(K^11^)**	C_91_H_140_N_24_O_26_S_2_	684.3355 [M+3H]^3+^	684.3349	≥98.2
**NODA-GA-TJ12P2(K^11^)**	C_75_H_127_N_25_O_26_	598.9868 [M+3H]^3+^	598.9865	≥98.5
**TJ12P2(K^11^)-X_3_K(NODA-GA)-CONH_2_**	C_90_H_155_N_31_O_29_	712.3942 [M+3H]^3+^	712.3936	≥98.6
***Scr*** **-TJ12P2(K^11^)**	C_60_H_104_N_22_O_19_	479.9356 [M+3H]^3+^	479.9354	≥99.0
**TJ12P2(Q^11^)**	C_59_H_100_N_22_O_20_	719.3815 [M+2H]^2+^	719.3813	≥99.1
**SulfoCy3-TJ12P2(Q^11^)**	C_90_H_136_N_24_O_27_S_2_	684.3233 [M+3H]^3+^	684.3229	≥99.8
**NODA-GA-TJ12P2(Q^11^)**	C_74_H_123_N_25_O_27_	598.9746 [M+3H]^3+^	598.9744	≥96.4
**TJ12P2(Q^11^)-X_3_K(NODA-GA)-CONH_2_**	C_89_H_151_N_31_O_30_	712.7165 [M+3H]^3+^	712.7159	≥98.5
***Scr*** **-TJ12P2(Q^11^)**	C_59_H_100_N_22_O_20_	719.3815 [M+2H]^2+^	719.3812	≥99.0

**Table 2:** Radiochemical conversion and molar activities of NODA-GA-bearing peptides labeled with [^67^Ga]GaCl_3_ in NH_4_OAc buffer (pH = 4.5) at 60 °C for 30 min as well as Log*D*_7.4_ values of the resulting radioligands.

Peptide	RCC [%]	A_M_ [MBq/nmol]	Log*D*_7.4_
**[^67^Ga]Ga-NODA-GA-TJ12P2(K^11^)**	98.8 ± 0.6 (n = 3)	5.5 ± 0.5 (n = 3)	−4.78 ± 0.05 (n = 4)
**TJ12P2(K^11^)-X_3_K-[^67^Ga]Ga-NODA-GA-CONH_2_**	99.3 ± 0.6 (n = 3)	5.9 ± 0.8 (n = 3)	−4.79 ± 0.03 (n = 4)
**[^67^Ga]Ga-NODA-GA-TJ12P2(Q^11^)**	99.7	5.13	−4.79 ± 0.03 (n = 4)
**TJ12P2(Q^11^)-X_3_K-[^67^Ga]Ga-NODA-GA-CONH_2_**	99.7	3.30	−4.61 ± 0.05 (n = 4)

## Data Availability

The original contributions presented in this study are included in the article/Supplementary Materials. Further inquiries can be directed to the corresponding author.

## Supplementary Materials

The following supporting information can be downloaded at: https://www.mdpi.com/article/10.3390/ph18111656/s1. Peptide synthesis, purification and characterization; Nanobody synthesis; Additional Information.

## Abbreviations

The following abbreviations are used in this manuscript:

Alloc	Allyloxycarbonyl
DBCO	Dibenzocyclooctin
BCN	Bicyclo[6.1.0]non-4-yne
Df	Deferoxamine
DTT	Dithiothreitol
DMSO	Dimethyl sulfoxide
EDTA	Ethylenediaminetetraacetic acid
ELISA	Enzyme-linked immunosorbent assay
FC	Flow cells
FDG	Fluordesoxyglucose
Fmoc	Fluorenylmethoxycarbonyl
GPC3	Glypican-3
HCC	Hepatocellular carcinoma
HPLC	High-performance liquid chromatography
HR	High resolution
MALDI	Matrix-assisted laser desorption/ionization
MS	Mass spectrometry
NODA-GA	1,4,7-triazacyclononane-1-glutaric acid-4,7-diacetic acid
NOTA	1,4,7-Triazacyclononane-1,4,7-triacetic acid
PAGE	Polyacrylamide gel electrophoresis
PBS	Phosphate-buffered saline
PET	Positron emission tomography
PMSF	Phenylmethylsulfonyl fluoride
POD	Peroxidase
PVDF	Polyvinylidene fluoride
RIPA	Radioimmunoprecipitation assay buffer
RP	Reverse phase
SDS	Sodium dodecyl sulfate
SPR	Surface plasmon resonance
TLC	Thin layer chromatography
TOF	Time of flight
