# Supplementary material for: In Vitro Characterization of the Published Glypican-3-Targeting Peptide TJ12P2 Reveals a Lack of Specificity and Potency
Source: Pharmaceuticals (Basel). 2025 Nov 1;18(11):1656. doi: 10.3390/ph18111656 (PMC12655185; doi:10.3390/ph18111656)
Supplement: Supplementary file 1 [file pharmaceuticals-18-01656-s001.zip › pharmaceuticals-3935848-supplementary.pdf]

Supplementary materials

S1 Peptide synthesis, purification and characterization

All peptides were synthesized using standard solid phase Fmoc-chemistry. General procedures for distinct steps in peptide synthesis are described below. Steps I-IV and VII-IX were carried out manually in a filter-capped syringe. Steps V and VI were carried out in an automated synthesizer system equipped with a microwave (Biotage, Initiator+Alstra).

(I) Swelling of resins

2-CTC resin (1.6 mmol/mg, Iris biotech), Wang resin (0.7 mmol/mg, Iris biotech) or Rink amide resin (Fmoc-protected, carbolution, 0.722 µmol/mg) was swelled in DMF or DCM (1 mL/100 mg resin) at ambient temperature for 10 min. The liquid was discarded and the swelled resin was used for further synthesis.

(II) Loading and capping of 2-CTC resin

The swelled resin was taken up in DCM (1 mL/100 mg resin). Subsequently, the respective protected amino acid (2.00 eq) and DIPEA (4.00 eq) were added and the mixture shaken at ambient temperature for 2 h. Subsequently, the solution was discarded, and the resin was taken up in DCM/MeOH/DIPEA (17/2/1, v/v/v) to cap any leftover free binding sites. The mixture was shaken at ambient temperature for 1 h. After washing with DMF (3x), DCM (3x) and DMF (3x), the loaded resin was used in further synthesis.

(III) Loading of Rink amide resin

Swelled resin was taken up in DMF/Piperidine (80:20, 2 mL/100 mg resin). After shaking at ambient temperature for 10 min, the liquid was discarded, and the procedure was repeated once. Subsequently, the resin was washed with DMF (3x) and taken up in DMF (1 mL/100 mg resin). Fmoc-L-Lys(Alloc)-OH (4.00 eq), HATU (4.00 eq) and DIPEA (8.00 eq) were added and the mixture was shaken at ambient temperature for 1 h. After discarding the solution, the resin was washed with DMF (3x), DCM (3x) and DMF (3x). The coupling procedure was repeated twice in total. Then, the resin was taken up in DMF (1 mL/100 mg resin) again and Ac_2O (10.0 eq) and DIPEA (20.0 eq) were added. After shaking for 45 min at ambient temperature, the liquid was removed and the capped resin washed with DMF (3x), DCM (3x) and DMF (3x). The loaded resin was used in further synthesis.

(IV) Loading and capping of Wang resin

Swelled resin was taken up in DMF (1 mL/100 mg resin). Subsequently, the respective Fmoc-protected amino acid (4.00 eq), DIC (4.00 eq), Oxyma (4.00 eq) and DIPEA (8.00 eq) were added, and the mixture was shaken at ambient temperature for 1 h. The resin was washed with DMF twice, and the loading procedure was repeated once. After washing with DMF, residual free hydroxy groups were capped using Ac_2O (10.0 eq) and DIPEA (20.0 eq) in DMF at ambient temperature for 30 min. The resin was washed with DMF (3x), DCM (3x) and DMF (3x). The loaded resin was used in further synthesis.

(V) Coupling of standard amino acids

To the swelled resin, the respective protected amino acid (4.00 eq, 0.25 M in DMF), HATU (4.00 eq, 0.5 M in DMF) and DIPEA (8.00 eq, 2 M in NMP) were added. Coupling of the amino acids was carried out using microwave heating at 75 °C and oscillating mixing for 5 min (Rink amide resin) or 40 °C and oscillating

mixing for 60 min (2-CTC resin). For Fmoc-L-Arg(Pbf)-OH, the reaction was performed without microwave heating at ambient temperature for 60 min. Afterwards, the resin was washed twice with DMF.

(VI) Fmoc removal

Fmoc was removed by incubating the resin with 20% piperidine in DMF (2 mL/100 mg resin) at ambient temperature for 10 min twice. After the introduction of aspartate into the peptides, HOBt was added to the deprotection mixture (0.1 M final solution) for all subsequent steps to prevent aspartimide formation.

(VII) Alloc removal

The dry resin was swelled in DCM (1 mL/100 mg resin) for 10 min. After discarding the liquid, the swelled resin was taken up in DCM (1.5 mL/100 mg resin) and phenylsilane (15.0 eq) and $Pd(PPh_3)_4$ (0.25 eq) were added. The mixture was shaken at 40 °C for 15 min, after which the liquid was discarded. The procedure was repeated three times in total. After deprotection was completed, the resin was washed with sodium diethylcarbamate trihydrate solution (1 g/50 mL in DMF). This was achieved by adding the solution to the syringe and shaking at ambient temperature for 10 min. The procedure was repeated twice, and the resin was subsequently washed with DMF (3x), DCM (3x) and DMF (3x).

(VIII) Coupling of $(tBu)_3$-(R)-NODA-GA

The swelled resin was taken up in DMF (1 mL/100 mg) and $(t\text{Bu})_3$-(R)-NODA-GA (1.00 eq), HATU (1.10 eq) and DIPEA (2.00 eq) were added. The mixture was shaken at ambient temperature for 1.5 h. After discarding the liquid, the resin was washed with DMF (3x), DCM (3x), MeOH (3x) and Et_2O (3x). Subsequently, the resin was dried at 50 °C for 1.5 h.

(IX) Global deprotection + cleavage from resin

To the dried resin were added H_2O (25 µL/100 mg resin), TIPS (25 µL/100 mg resin) and TFA (950 µL/100 mg resin). The mixture was shaken at 40 °C for 1 h. After complete cleavage, the TFA-solution was collected in a 50 mL falcon tube and the resin was washed with TFA (500 µL/100 mg resin) twice. Cold Et_2O (−20 °C, 10 mL/1 mL TFA solution) was added to the TFA solution and the mixture was left to stand at −20 °C for at least 1 h. After centrifuging (7000 rpm) at 4 °C for 10 min, the ether was carefully discarded and the precipitate was washed with cold ether and centrifuged twice, as previously described. Drying for 1.5 h at 50 °C yielded the respective crude peptides.

Peptide purification and analysis:

Crude peptides were purified using a modular HPLC system by Shimadzu (LC-20A Prominance) which consists of two separate pumping units (LC-A20R), a degasser (DGU-20A5R), an autosampler (SIL-20ACHT), a column oven (CTO-20AC), a PDA detector (SPD-M20A), a fraction collector (FRC-10A) and a system controller unit (CBM-M20A). For semipreparative and analytical HPLC, Aeris Peptide CB-C18 columns (100 Å pore size, 250 x 21.2 mm or 250 x 4.6 mm) from Phenomenex were used, and columns were heated to 50 °C by default. Water and acetonitrile mixed with 0.1% TFA were used as eluents in the specified gradients, which are given as [percentage of water in mixture, W]:[percentage of MeCN in mixture, A]. Flow rates of 10 mL/min were employed for semipreparative HPLC, and employed methods are based on the following scheme with W1:A1 and W2:A2 specified for each compound.

W1:A1 5 min; W1:A1 → W2:A2 20 min; W2:A2 → 5:95 1 min; 5:95 5 min; 5:95 → W1:A1 1 min; W1:A1 5 min.

Analytical HPLC chromatograms were recorded using a 1 mL/min flow rate, and the employed method is described here:

95:5 5 min; 95:5 → 45:55 25 min; 45:55 → 5:95 1 min; 5:95 3 min; 5:95 → 95:5 1 min; 95:5 12 min.

All depicted analytical chromatograms show the absorption at 220 nm. All depicted chromatograms were normalized. Applied methods are visualized below:

HRMS spectra were recorded on a Q-TOF mass spectrometer using electrospray ionization. The mass spectrometer consists of an Agilent 1260 Infinity II HPLC (Santa Clara, California, USA; pump G7104C, autosampler G7129C, column oven G7116A, DAD detector G7117C) coupled to a γ-detector Gabi Star (Raytest Isotopenmeßgeräte GmbH, Straubenhardt, Germany) and mass detection unit Revident Q-TOF LC/Q-TOF G6575A. Measurements were performed in bypass mode using an eluent consisting of MeCN and 0.1% formic acid in H_2O at a flow rate 0.2 mL/min. Referencing was carried out by co-injection of a solution containing hexakis(1H,1H,3H-tetrafluoropropoxy)phosphazene and purine *via* dual AJS ESI source. Operation of the system occurred by an Agilent Masshunter Workstation 3.6 – LC/MS data aquisition software (Version 12.0) and data evaluation was performed using Agilent Masshunter Workstation 3.6 Qualitative Analysis software (Version 12.0 Update 1).

The peptides **TJ12P2(Q¹¹)**, **TJ12P2(K¹¹)**, **NODA-GA-TJ12P2(Q¹¹)**, **NODA-GA-TJ12P2(K¹¹)**, ***scr*-TJ12P2(Q¹¹)**, ***scr*-TJ12P2(K¹¹)**, **TJ12P2(Q¹¹)-X₃K(NODA-GA)-CONH₂** and **TJ12P2(K¹¹)-X₃K(NODA-GA)-CONH₂** were synthesized according to the following schemes. (a) Standard peptide coupling and Fmoc removal conditions; reactions were carried out as described in general procedures (V), (VI) and (VIII); (b) TFA:H_2O:TIPS (95:2.5:2.5), 40 °C, 1 h; precise conditions are described in general procedures (IX); (c) $Pd(PPh_3)_4$ (0.25 eq), $PhSiH_3$ (15.0 eq), DCM, 40 °C, 15 min; precise conditions are described in general procedures (VII).

Synthesis of ***TJ12P2(K¹¹)****,* ***SulfoCy3-TJ12P2(K¹¹)*** *and* ***NODA-GA TJ12P2(K¹¹)****,* ***TJ12P2(Q¹¹)****,* ***SulfoCy3-TJ12P2(Q¹¹)*** *and* ***NODA-GA TJ12P2(Q¹¹)****:*

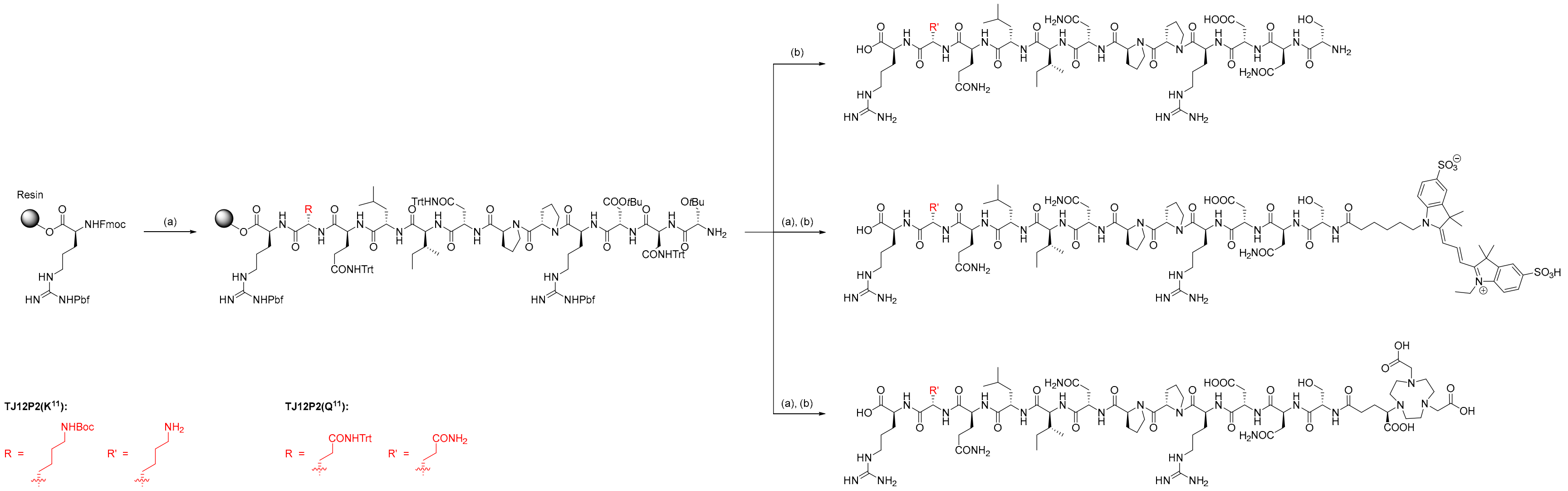

Figure S1: Synthetic scheme for solid phase synthesis of **TJ12P2(K¹¹)**, **SulfoCy3-TJ12P2(K¹¹)**, **NODA-GA TJ12P2(K¹¹)**, **TJ12P2(Q¹¹)**, **SulfoCy3-TJ12P2(Q¹¹)** and **NODA-GA TJ12P2(Q¹¹)** starting from the respective loaded 2-CTC resin.

Synthesis of ***scr-TJ12P2(K^{11})***:

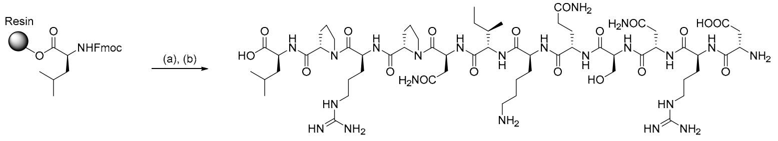

Figure S2: Synthetic scheme for solid phase synthesis of ***scr*-TJ12P2(K^{11})** starting from the respective loaded Wang resin.

Synthesis of ***scr-TJ12P2(Q^{11})***:

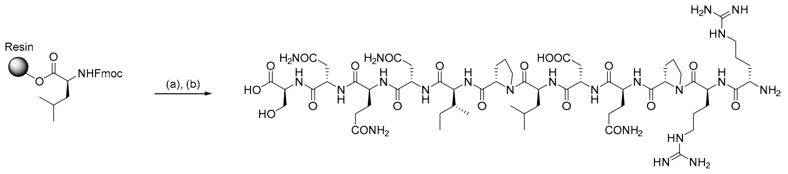

Figure S3: Synthetic scheme for solid phase synthesis of ***scr*-TJ12P2(Q^{11})** starting from the respective loaded 2-CTC resin.

Synthesis of ***TJ12P2(K^{11})-X$_3$K(NODA-GA)-CONH$_2$*** *and* ***TJ12P2(Q^{11})-X$_3$K(NODA-GA)-CONH$_2$***:

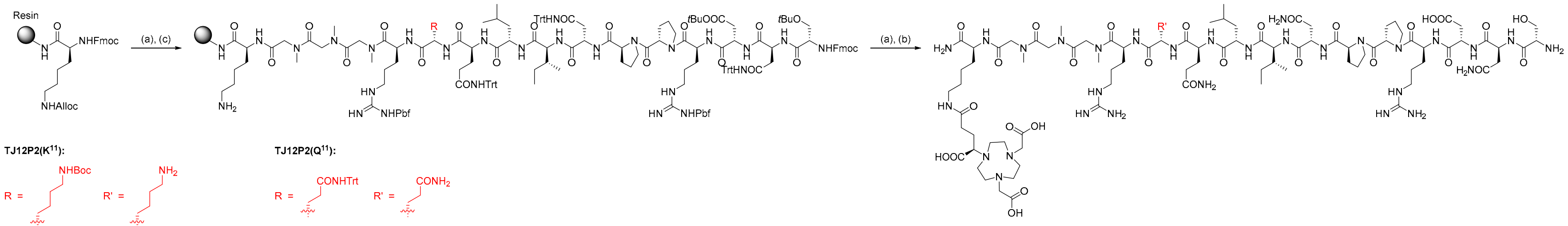

Figure S4: Synthetic scheme for solid phase synthesis of **TJ12P2(K^{11})-X$_3$K(NODA-GA)-CONH$_2$** and **TJ12P2(Q^{11})-X$_3$K(NODA-GA)-CONH$_2$** starting from the respective loaded Rink amide resin.

Synthesis of ***TJ12P2(K*** [11] ***)***

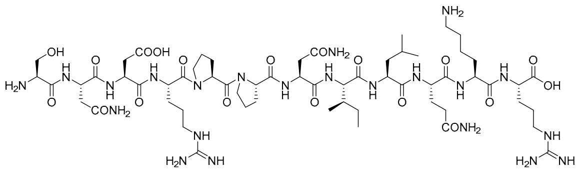

SNDRPPNILQKR

Peptide **TJ12P2(K[11])** was synthesized by standard Fmoc peptide chemistry from Wang resin (24.5 mg, 1.6 g/mmol, 39.1 µmol) as described in **Figure S1** above.

Preparative HPLC: W1:A1 → W2:A2 = 88:12 → 77:23; R_T = 20.5 min.
Yield: 10.5 mg (7.30 µmol, 19%) of a colorless solid.
Analytical HPLC: R_T = 18.5 min; Purity = 99.8% (220 nm).
HRMS: m/z calcd. for $C_{60}H_{104}N_{22}O_{19}$: 719.3997 $[M+2H]^{2+}$; found: 719.3994.

Synthesis of ***SulfoCy3-TJ12P2(K*** [11] ***)***

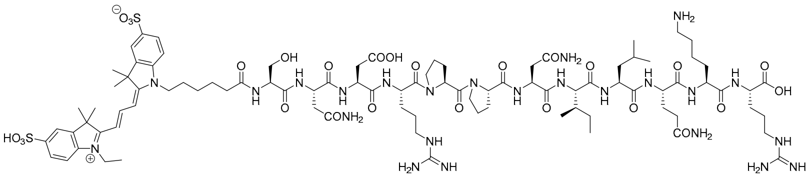

SulfoCy3-SNDRPPNILQKR

Peptide **SulfoCy3-TJ12P2(K[11])** was synthesized by standard Fmoc peptide chemistry from 2-CTC resin (16.3 mg, 1.6 g/mmol, 26.0 µmol) as described in **Figure S1** above.

Preparative HPLC: W1:A1 → W2:A2 = 90:10 → 68:32; R_T = 24.0 min.
Yield: 3.74 mg (1.82 µmol, 7%) of a pink solid.
Analytical HPLC: R_T = 21.1 min; Purity = 98.2% (220 nm).
HRMS: m/z calcd. for $C_{91}H_{140}N_{24}O_{26}S_2$: 684.3355 $[M+3H]^{3+}$; found: 684.3349.

Synthesis of ***NODA-GA-TJ12P2(K*** [11] ***)***

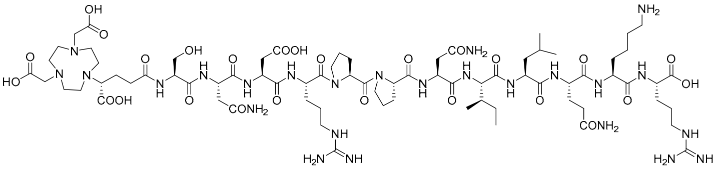

NODA-GA-SNDRPPNILQKR

Peptide **NODA-GA-TJ12P2(K[11])** was synthesized by standard Fmoc peptide chemistry from Wang resin (69.2 mg, 0.7 g/mmol, 48.5 µmol) as described in **Figure S1** above.

Preparative HPLC: W1:A1 → W2:A2 = 88:12 → 76:24; R_T = 21.5 min.

Yield: 19.5 mg (10.9 µmol, 22%) of a colorless solid.
Analytical HPLC: R_T = 18.6 min; Purity = 98.5% (220 nm).
HRMS: m/z calcd. for $C_{75}H_{127}N_{25}O_{26}$: 598.9868 $[M+3H]^{3+}$; found: 598.9865.

Synthesis of ***scr-TJ12P2(K[11])***

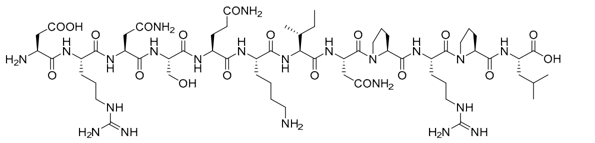

DRNSQKINPRPL

Peptide ***scr*-TJ12P2(K[11])** was synthesized by standard Fmoc peptide chemistry from Wang resin (49.6 mg, 0.7 g/mmol, 34.7 µmol) as described in **Figure S2:** above.

Preparative HPLC: W1:A1 → W2:A2 = 90:10 → 71:29; R_T = 20.0 min.
Yield: 12.4 mg (8.63 µmol, 25%) of a colorless solid.
Analytical HPLC: R_T = 18.0 min; Purity = 99.0% (220 nm).
HRMS: m/z calcd. for $C_{60}H_{104}N_{22}O_{19}$: 479.9356 $[M+3H]^{3+}$; found: 479.9354.

Synthesis of ***TJ12P2(K[11])-X$_3$K(NODA-GA)-CONH$_2$***

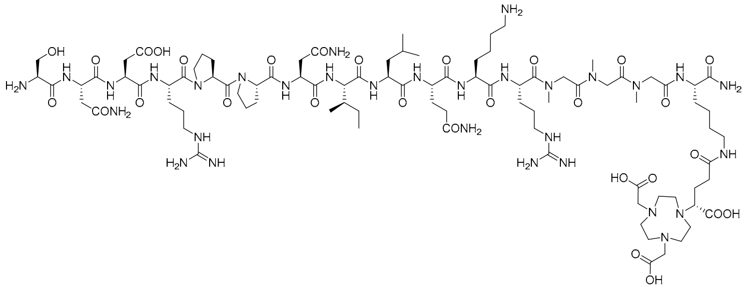

SNDRPPNILQKRXXXK(NODA-GA)-CONH$_2$

Peptide **TJ12P2(K[11])-X$_3$K(NODA-GA)-CONH$_2$** was synthesized by standard Fmoc peptide chemistry from Rink amide resin (40.0 mg, 0.14 g/mmol, 5.60 µmol) as described in **Figure S4** above.

Preparative HPLC: W1:A1 → W2:A2 = 88:12 → 77:23; R_T = 21.9 min.
Yield: 2.5 mg (1.06 µmol, 19%) of a colorless solid.
Analytical HPLC: R_T = 18.3 min; Purity = 98.6% (220 nm).
HRMS: m/z calcd. for $C_{90}H_{155}N_{31}O_{29}$: 712.3942 $[M+3H]^{3+}$; found: 712.3936.

Synthesis of ***TJ12P2(Q11)***

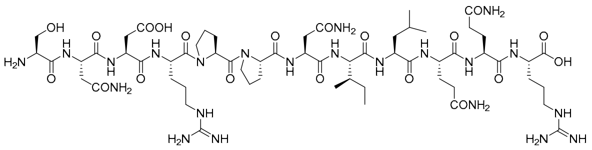

SNDRPPNILQQR

Peptide **TJ12P2(Q11)** was synthesized by standard Fmoc peptide chemistry using 2-CTC resin (16.4 mg, 1.6 g/mmol, 26.3 μmol) as described in **Figure S1** above.

Preparative HPLC: W1:A1 → W2:A2 = 95:5 → 76:24; R_T = 21.9 min.
Yield: 7.96 mg (5.54 μmol, 21%) of a colorless solid.
Analytical HPLC: R_T = 18.9 min; Purity = 99.1% (220 nm).
HRMS: m/z calcd. for $C_{59}H_{100}N_{22}O_{20}$: 719.3815 $[M+2H]^{2+}$; found: 719.3813.

Synthesis of ***SulfoCy3-TJ12P2(Q11)***

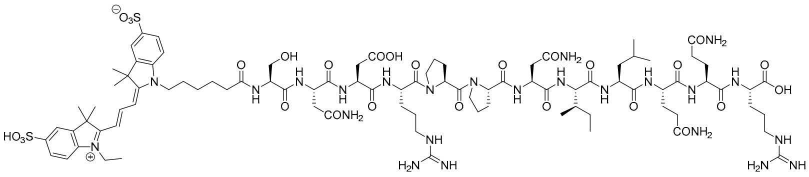

SulfoCy3-SNDRPPNILQQR

Peptide **SulfoCy3-TJ12P2(Q11)** was synthesized by standard Fmoc peptide chemistry from 2-CTC resin (8.81 mg, 1.6 g/mmol, 14.1 μmol) as described in **Figure S1** above.

Preparative HPLC: W1:A1 → W2:A2 = 85:15 → 71:29; R_T = 23.6 min.
Yield: 2.08 mg (1.01 μmol, 7%) of a pink solid.
Analytical HPLC: R_T = 21.4 min; Purity = 99.8% (220 nm).
HRMS: m/z calcd. for $C_{90}H_{136}N_{24}O_{27}S_2$: 684.3233 $[M+3H]^{3+}$; found: 684.3229.

Synthesis of ***NODA-GA-TJ12P2(Q11)***

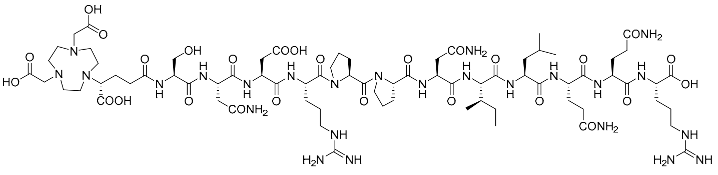

NODA-GA-SNDRPPNILQQR

Peptide **NODA-GA-TJ12P2(Q11)** was synthesized by standard Fmoc peptide chemistry from 2-CTC resin (34.9 mg, 1.6 g/mmol, 55.8 μmol) as described in **Figure S1** above.

Preparative HPLC: W1:A1 → W2:A2 = 90:10 → 74:26; R_T = 19.3 min.
Yield: 16.0 mg (8.93 μmol, 16%) of a colorless solid.

Analytical HPLC: R_T = 19.3 min; Purity = 96.4% (220 nm).
HRMS: m/z calcd. for $C_{74}H_{123}N_{25}O_{27}$: 598.9746 $[M+3H]^{3+}$; found: 598.9744.

*Synthesis of **scr-TJ12P2(Q[11])***

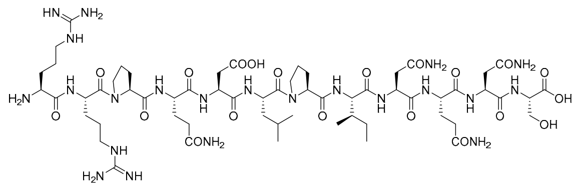

RRPQDLPINQNS

Peptide ***scr*-TJ12P2(Q[11])** was synthesized by standard Fmoc peptide chemistry from 2-CTC resin (9.12 mg, 1.6 g/mmol, 14.6 μmol) as described in **Figure S3:** above.

Preparative HPLC: W1:A1 → W2:A2 = 90:10 → 80:20; R_T = 23.5 min.
Yield: 11.3 mg (7.89 μmol, 54%) of a colorless solid.
Analytical HPLC: R_T = 17.7 min; Purity = 99.0% (220 nm).
HRMS: m/z calcd. for $C_{59}H_{100}N_{22}O_{20}$: 719.3815 $[M+2H]^{2+}$; found: 719.3812.

*Synthesis of **TJ12P2(Q[11])-X$_3$K(NODA-GA)-CONH$_2$***

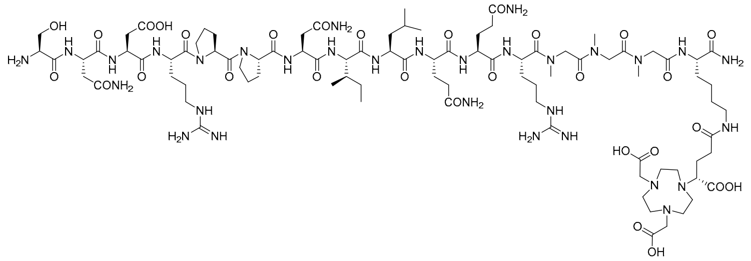

SNDRPPNILQQRXXXK(NODA-GA)

Peptide **TJ12P2(Q[11])-X$_3$K(NODA-GA)-CONH$_2$** was synthesized by standard Fmoc peptide chemistry from Rink amide resin (17.2 mg, 0.72 g/mmol, 12.4 μmol) as described in **Figure S4** above.

Preparative HPLC: W1:A1 → W2:A2 = 88:12 → 76:24; R_T = 22.4 min.
Yield: 5.26 mg (2.23 μmol, 18%) of a colorless solid.
Analytical HPLC: R_T = 18.9 min; Purity = 98.4% (220 nm).
HRMS: m/z calcd. for $C_{89}H_{151}N_{31}O_{30}$: 712.7165 $[M+3H]^{3+}$; found: 712.7159.

HPLC Chromatograms

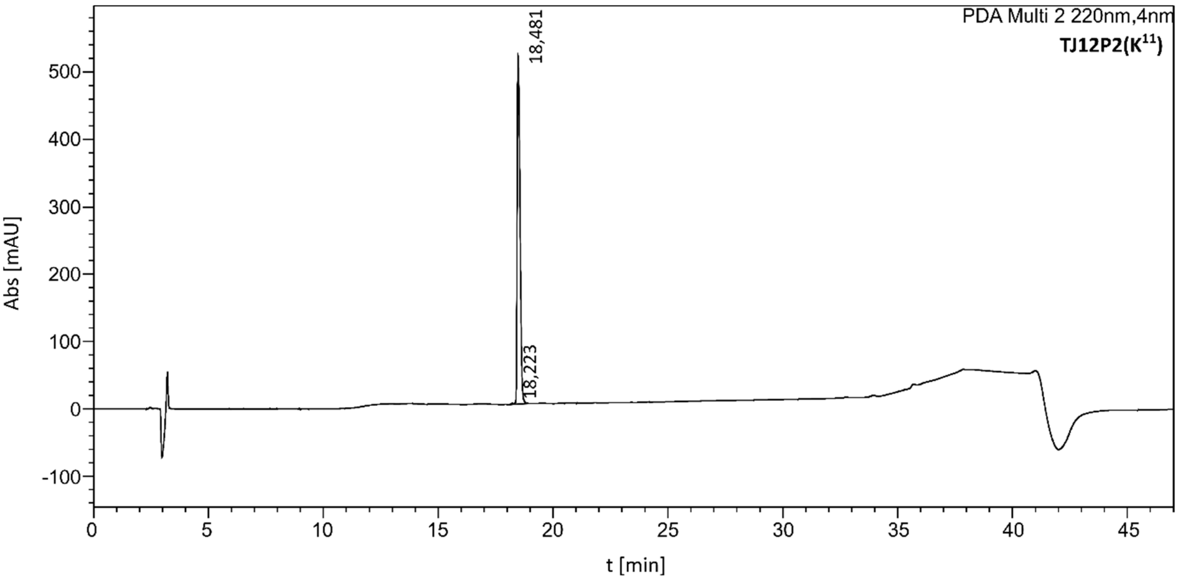

PDA Ch2 220nm

Peak#	Ret. Time	Area	Height	Area%
1	18,223	9781	1642	0,216
2	18,481	4512925	515421	99,784
Total		4522706	517063	100,000

Figure S5: Analytical HPLC chromatogram of **TJ12P2(K[11])** at 220 nm. The peptide had a purity of 99.8%.

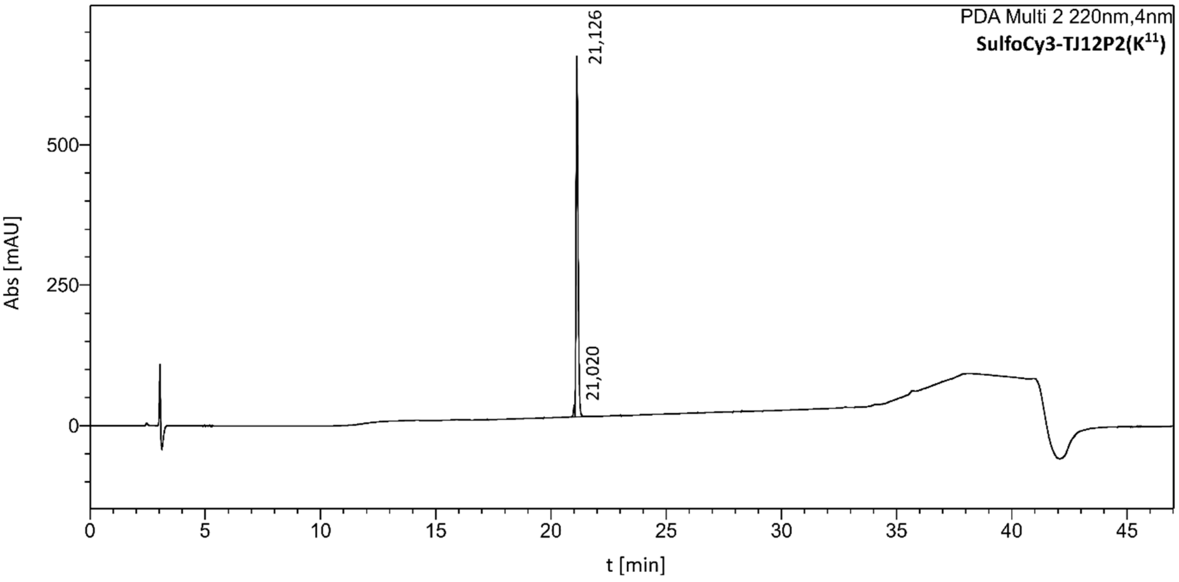

PDA Ch2 220nm

Peak#	Ret. Time	Area	Height	Area%
1	21,020	71493	20271	1,829
2	21,126	3837172	641074	98,171
Total		3908665	661345	100,000

Figure S6: Analytical HPLC chromatogram of **SulfoCy3-TJ12P2(K[11])**. The peptide had a purity of 98.2%.

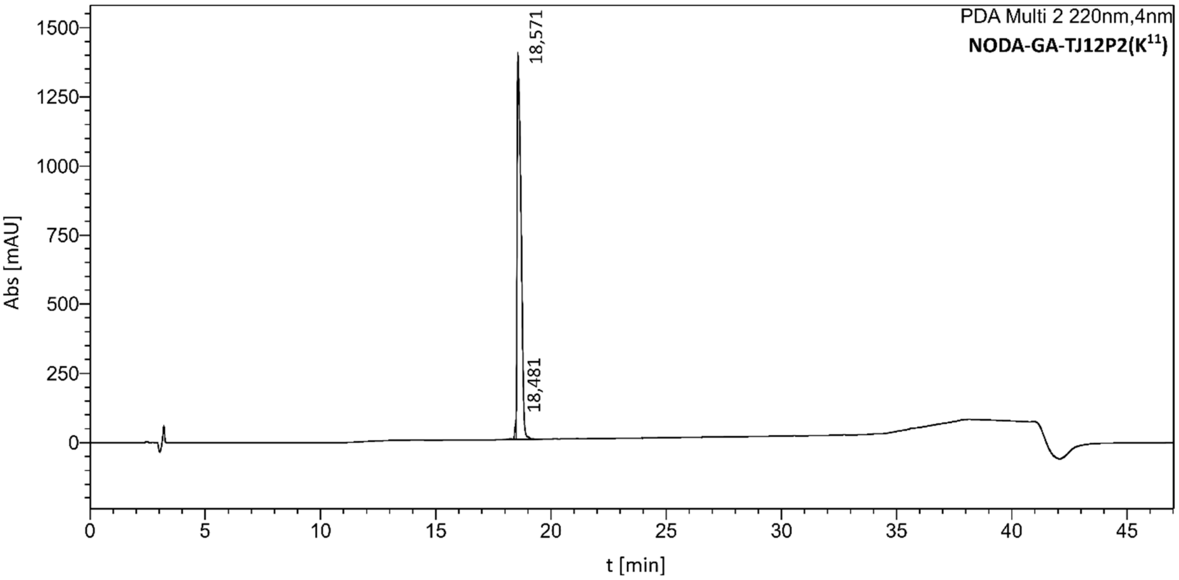

PDA Ch2 220nm

Peak#	Ret. Time	Area	Height	Area%
1	18,481	256529	60907	1,541
2	18,571	16391832	1386001	98,459
Total		16648361	1446908	100,000

Figure S7: Analytical HPLC chromatogram of **NODA-GA-TJ12P2(K[11])**. The peptide had a purity of 98.5%.

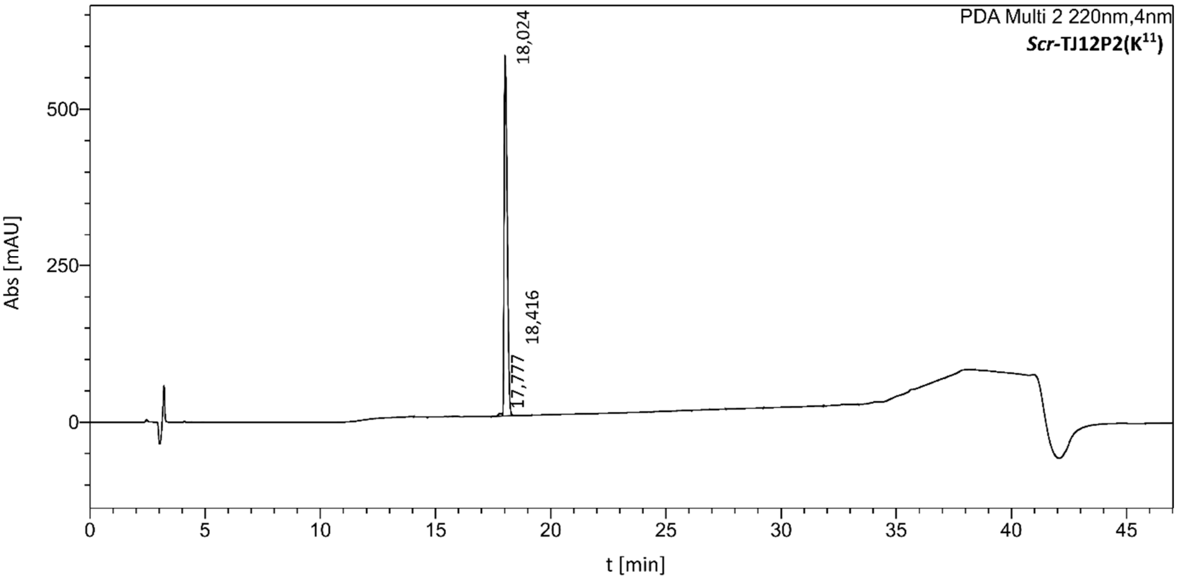

PDA Ch2 220nm

Peak#	Ret. Time	Area	Height	Area%
1	17,777	44148	4543	0,819
2	18,024	5335251	574374	98,976
3	18,416	11073	955	0,205
Total		5390471	579872	100,000

Figure S8: Analytical HPLC chromatogram of ***scr*-TJ12P2(K[11])**. The peptide had a purity of 99.0%.

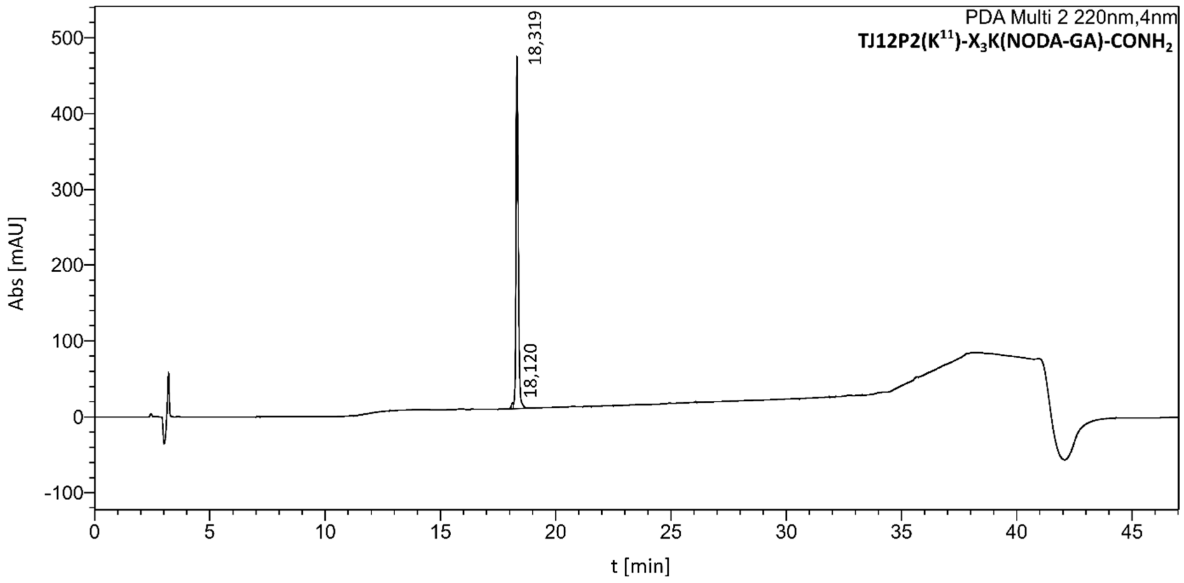

PDA Ch2 220nm

Peak#	Ret. Time	Area	Height	Area%
1	18,120	44485	7649	1,385
2	18,319	3167242	463588	98,615
Total		3211727	471237	100,000

Figure S9: Analytical HPLC chromatogram of **TJ12P2(K[11])-X$_3$K(NODA-GA)-CONH$_2$**. The peptide had a purity of 98.6%.

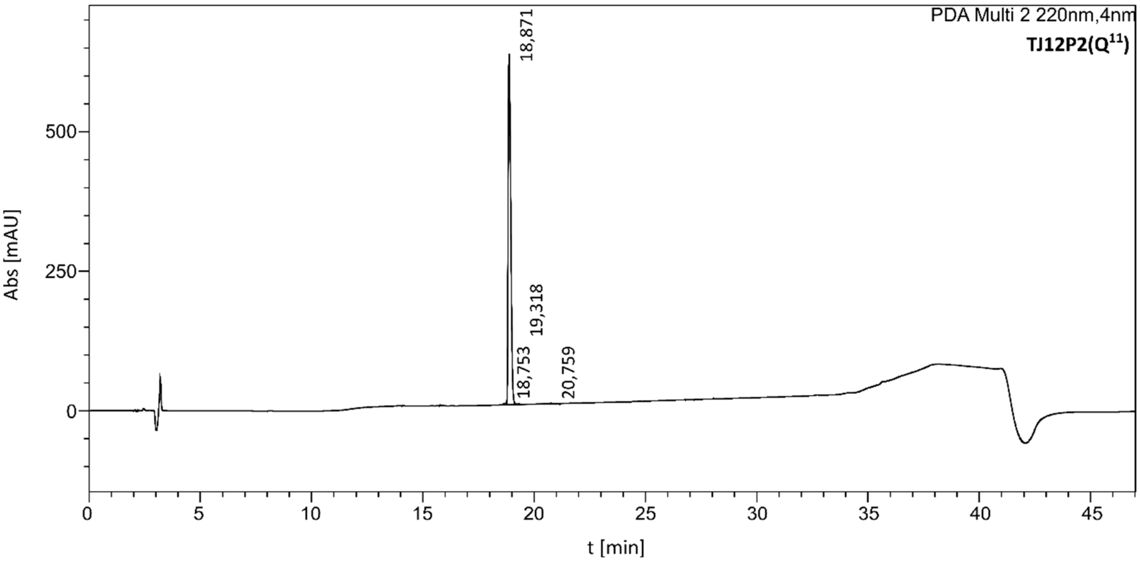

PDA Ch2 220nm

Peak#	Ret. Time	Area	Height	Area%
1	18,753	25976	3262	0,495
2	18,871	5198489	628220	99,129
3	19,318	14049	1289	0,268
4	20,759	5657	897	0,108
Total		5244171	633667	100,000

Figure S10: Analytical HPLC chromatogram of **TJ12P2(Q[11])** at 220 nm. The peptide had a purity of 99.1%.

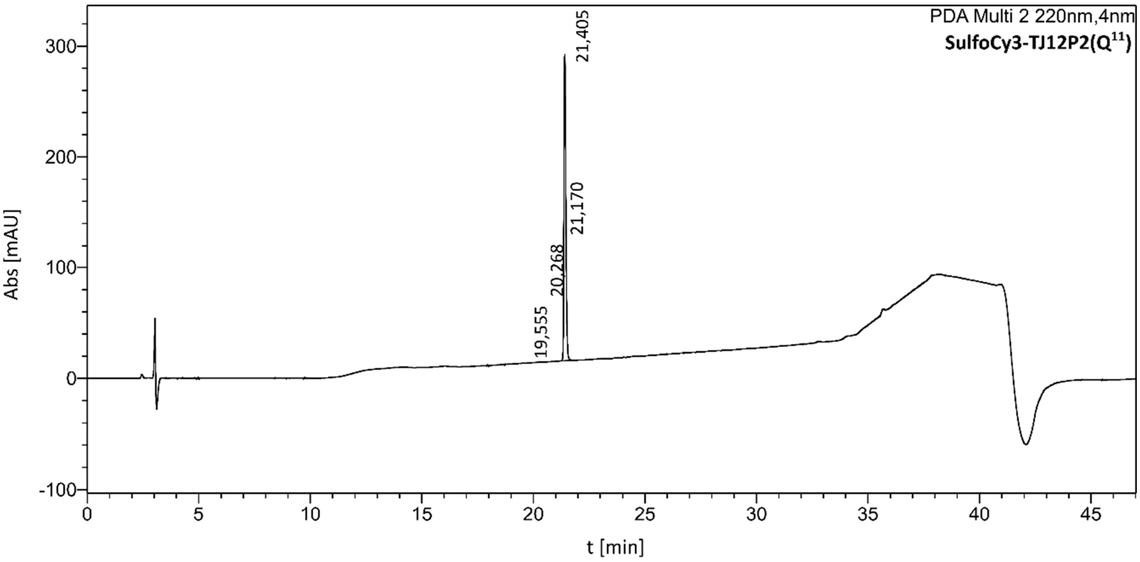

PDA Ch2 220nm

Peak#	Ret. Time	Area	Height	Area%
1	19,555	1297	163	0,079
2	20,268	1224	149	0,074
3	21,170	722	140	0,044
4	21,405	1642305	276167	99,803
Total		1645549	276619	100,000

Figure S11: Analytical HPLC chromatogram of **SulfoCy3-TJ12P2(Q[11])** at 220 nm. The peptide had a purity of 99.8%.

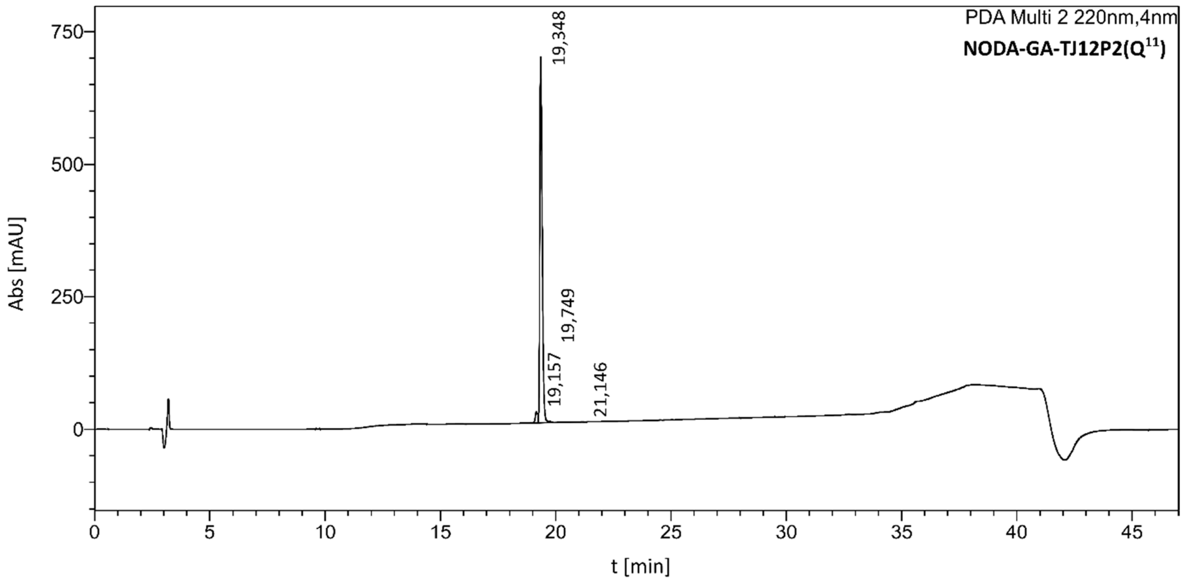

PDA Ch2 220nm

Peak#	Ret. Time	Area	Height	Area%
1	19,157	169021	21169	3,183
2	19,348	5117578	690276	96,380
3	19,749	22394	2313	0,422
4	21,146	821	339	0,015
Total		5309815	714096	100,000

Figure S12: Analytical HPLC chromatogram of **NODA-GA-TJ12P2(Q[11])** at 220 nm. The peptide had a purity of 96.4%.

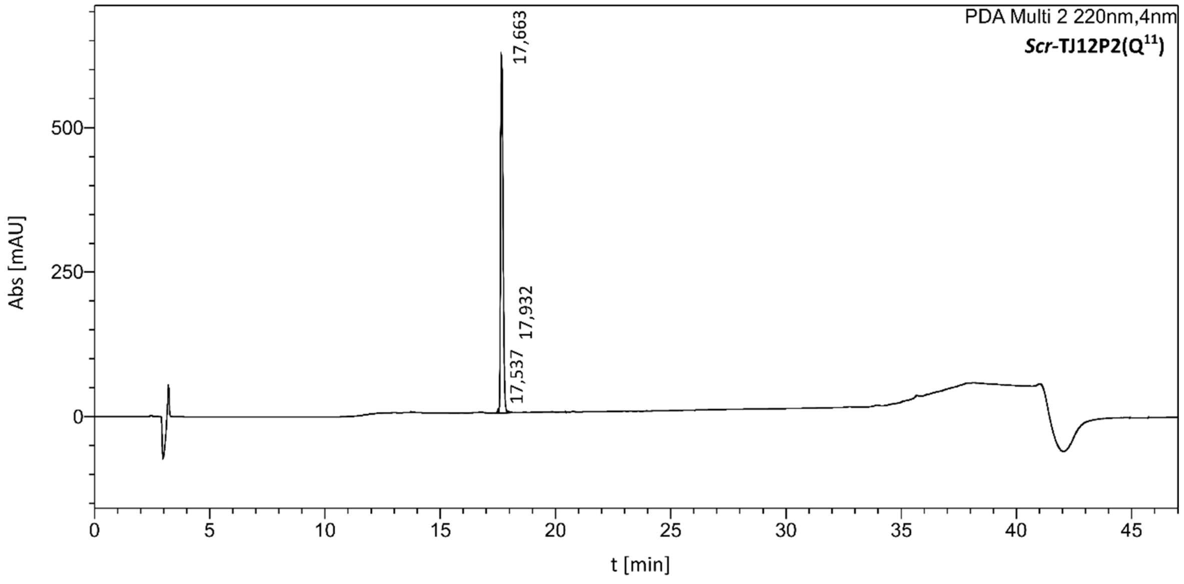

PDA Ch2 220nm

Peak#	Ret. Time	Area	Height	Area%
1	17,537	28078	7789	0,595
2	17,663	4671796	617186	98,959
3	17,932	21081	2964	0,447
Total		4720955	627939	100,000

Figure S13: Analytical HPLC chromatogram of ***scr*-TJ12P2(Q[11])** at 220 nm. The peptide had a purity of 99.0%.

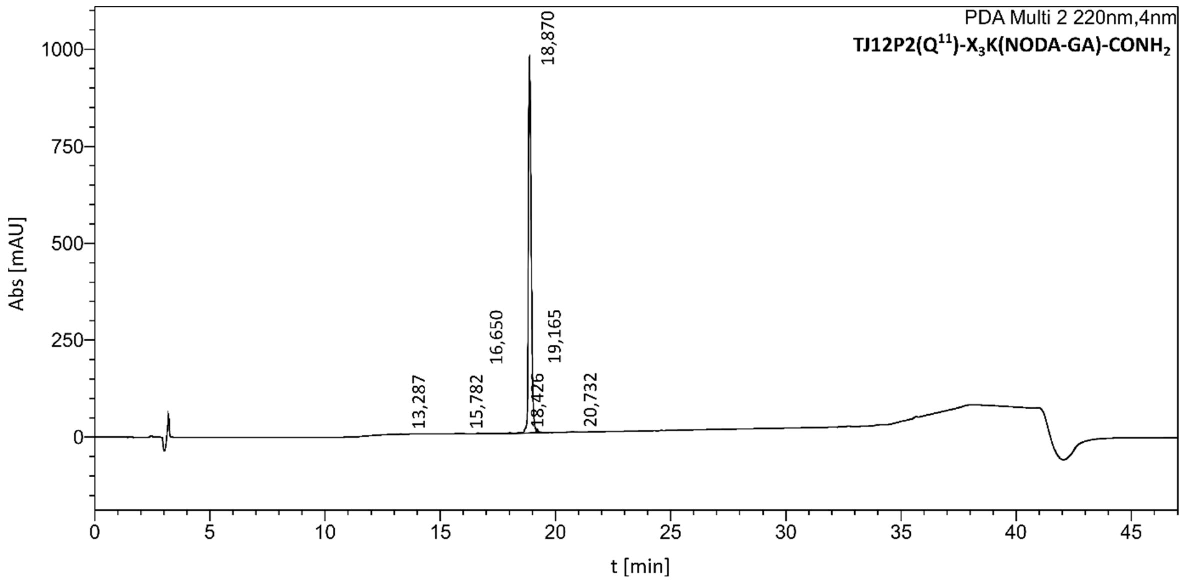

PDA Ch2 220nm

Peak#	Ret. Time	Area	Height	Area%
1	13,287	6101	1031	0,071
2	15,782	14270	642	0,167
3	16,650	12026	1870	0,141
4	18,426	27420	1994	0,321
5	18,870	8408043	968877	98,422
6	19,165	66652	10537	0,780
7	20,732	8306	1230	0,097
Total		8542817	986181	100,000

Figure S14: Analytical HPLC chromatogram of **TJ12P2(Q[11])-X$_3$K(NODA-GA)-CONH$_2$** at 220 nm. The peptide had a purity of 98.4%.

HRMS Spectra

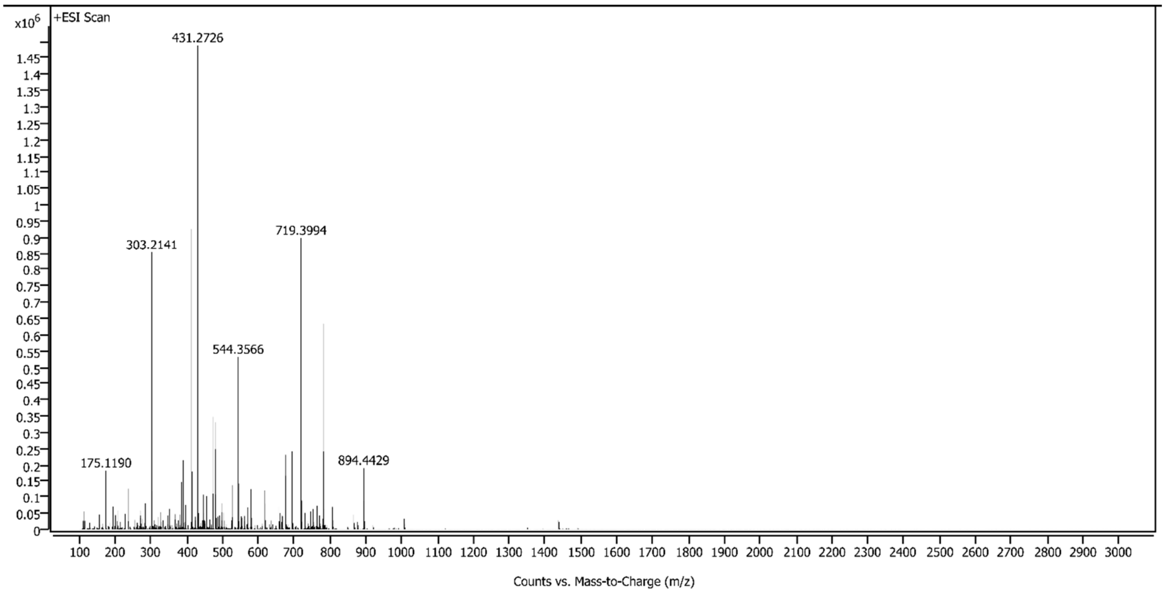

Spectrum Peaks

m/z	Z	Abund	Abund %	m/z (Calc)	Diff (ppm)	Ion Species	Formula
719.3994	2	897970	60.32	719.3997	0.42	$[M+2H]^{2+}$	$C_{60}H_{104}N_{22}O_{19}$
719.9009	2	673081	45.21				
720.4021	2	286547	19.25				
479.9354	3	330821	22.22				
480.2696	3	247784	16.64				

Figure S15: HRMS spectrum of **TJ12P2(K[11])**.

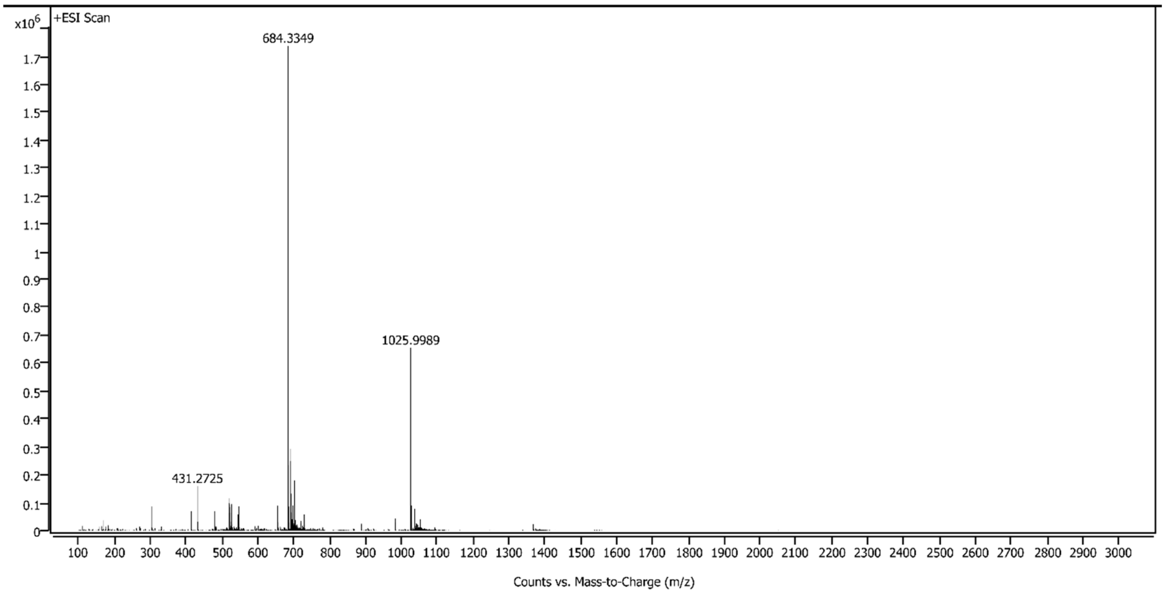

Spectrum Peaks

m/z	Z	Abund	Abund %	m/z (Calc)	Diff (ppm)	Ion Species	Formula
684.0006	3	1555396	89.48				
684.3349	3	1738300	100.00	684.3355	0.88	$[M+3H]^{3+}$	$C_{91}H_{140}N_{24}O_{26}S_2$
684.6689	3	1136800	65.40				
685.0026	3	572505	32.93				
685.3364	3	235812	13.57				
1025.4974	2	579984	33.36				
1025.9989	2	654210	37.64				
1026.4997	2	429774	24.72				
1027.0004	2	218963	12.60				

Figure S16: HRMS spectrum of **SulfoCy3-TJ12P2(K[11])**.

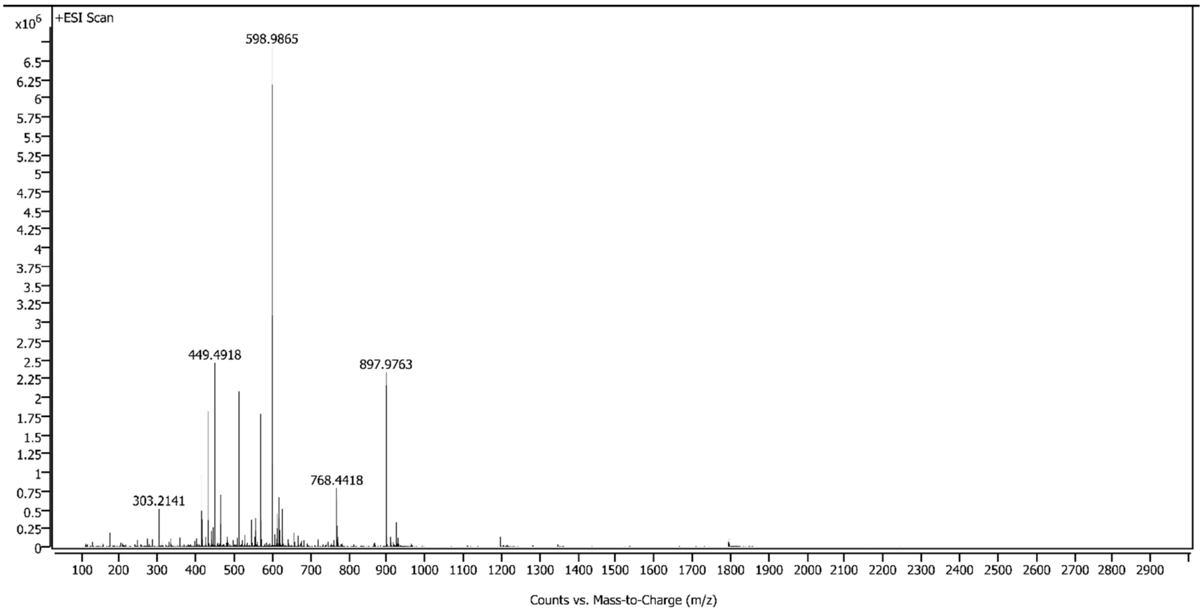

Spectrum Peaks

m/z	Z	Abund	Abund %	m/z (Calc)	Diff (ppm)	Ion Species	Formula
598.9865	3	6686148	100.00	598.9868	0.50	$[M+3H]^{3+}$	$C_{75}H_{127}N_{25}O_{26}$
599.3208	3	6182767	92.47				
599.6549	3	3096143	46.31				
599.9891	3	1114679	16.67				
897.9763	2	2337017	34.95				
898.4776	2	2164362	32.37				
898.9789	2	1081067	16.17				

Figure S17: HRMS spectrum of **NODA-GA-TJ12P2(K[11])**.

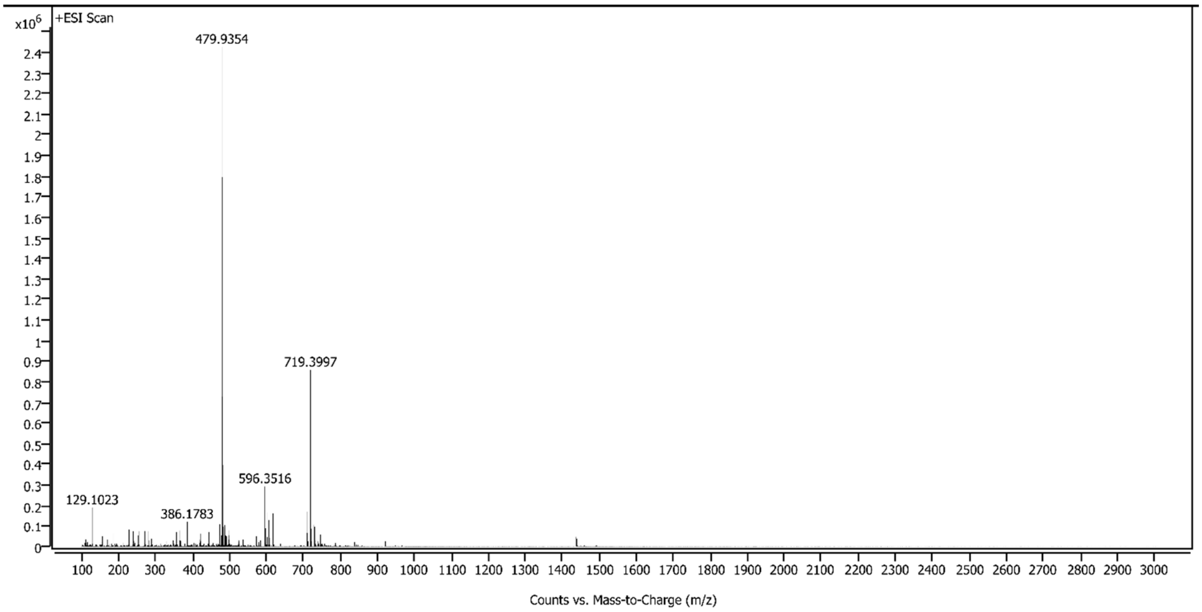

Spectrum Peaks

m/z	Z	Abund	Abund %	m/z (Calc)	Diff (ppm)	Ion Species	Formula
479.9354	3	2426036	100.00	479.9356	0.42	$[M+3H]^{3+}$	$C_{60}H_{104}N_{22}O_{19}$
480.2697	3	1794201	73.96				
480.6038	3	728297	30.02				
719.3997	2	859318	35.42				
719.9008	2	633745	26.12				
720.4021	2	276482	11.40				
720.9032	2	87873	3.62				

Figure S18: HRMS spectrum of ***scr*-TJ12P2(K[11])**.

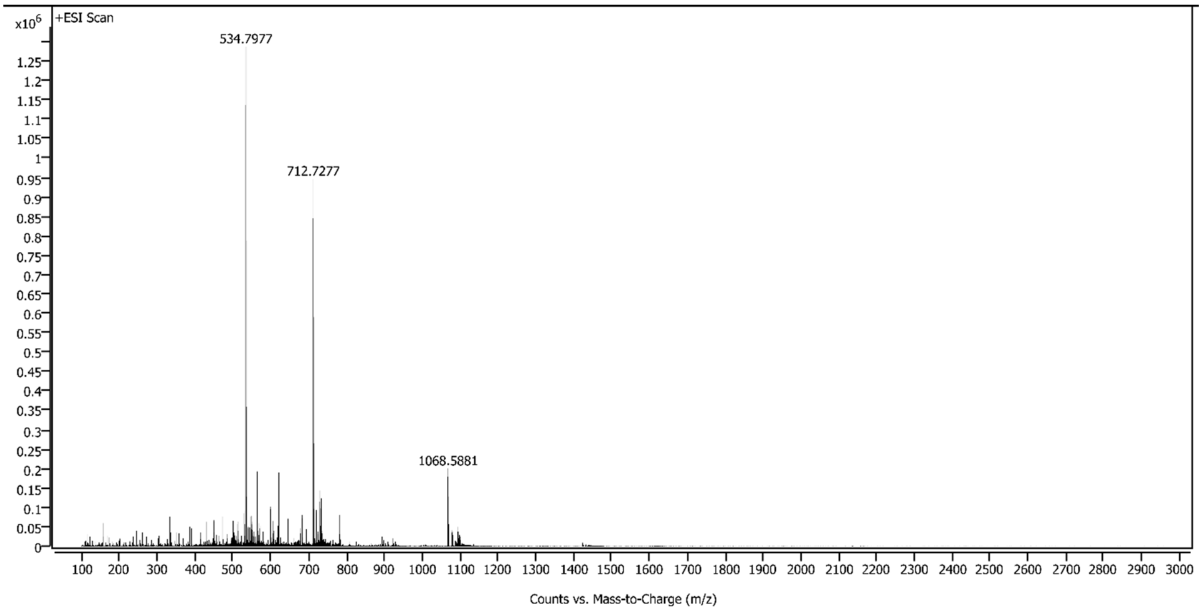

Spectrum Peaks

m/z	Z	Abund	Abund %	m/z (Calc)	Diff (ppm)	Ion Species	Formula
534.5471	4	1137027	88.35				
534.7977	4	1286993	100.00				
535.0482	4	787948	61.22				
535.2988	4	358830	27.88				
712.3936	3	845147	65.67	712.3942	1.00	$[M+3H]^{3+}$	$C_{90}H_{155}N_{31}O_{29}$
712.7277	3	946639	73.55				
713.0618	3	589248	45.78				
713.3959	3	265385	20.62				
1068.0868	2	179001	13.91				
1068.5881	2	199648	15.51				
1069.0894	2	127099	9.88				

Figure S19: HRMS spectrum of **TJ12P2(K[11])-X$_3$K(NODA-GA)-CONH$_2$**.

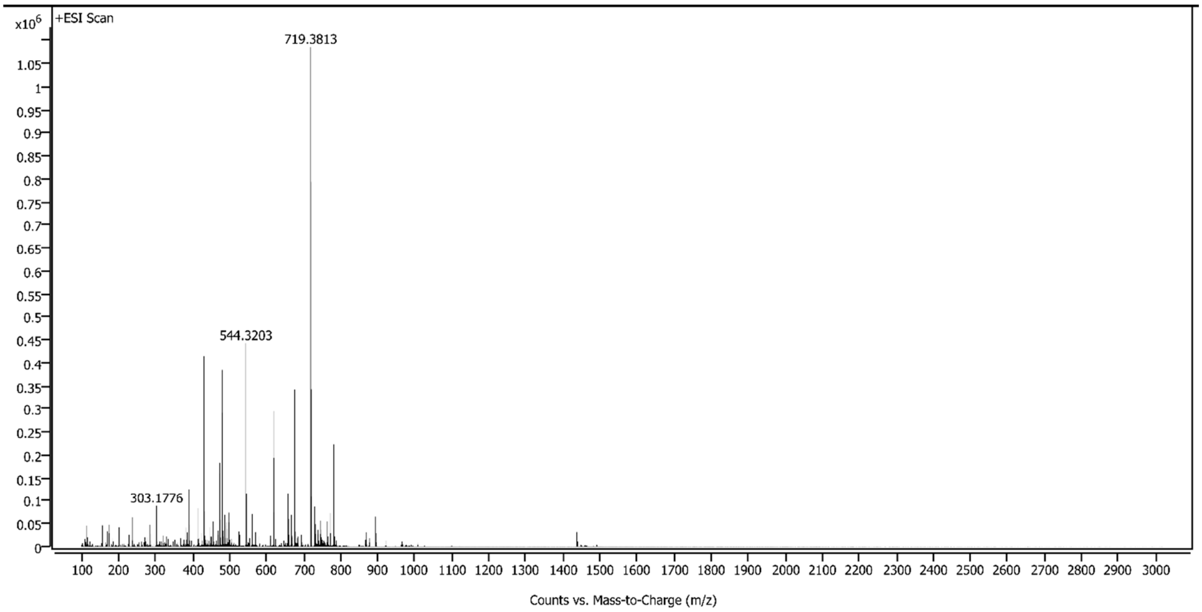

Spectrum Peaks

m/z	Z	Abund	Abund %	m/z (Calc)	Diff (ppm)	Ion Species	Formula
719.3813	2	1085479	100.00	719.3815	0.28	$[M+2H]^{2+}$	$C_{59}H_{100}N_{22}O_{20}$
719.8825	2	793226	73.08				
720.3837	2	343069	31.61				
720.8847	2	109778	10.11				

Figure S20: HRMS spectrum of **TJ12P2(Q[11])**.

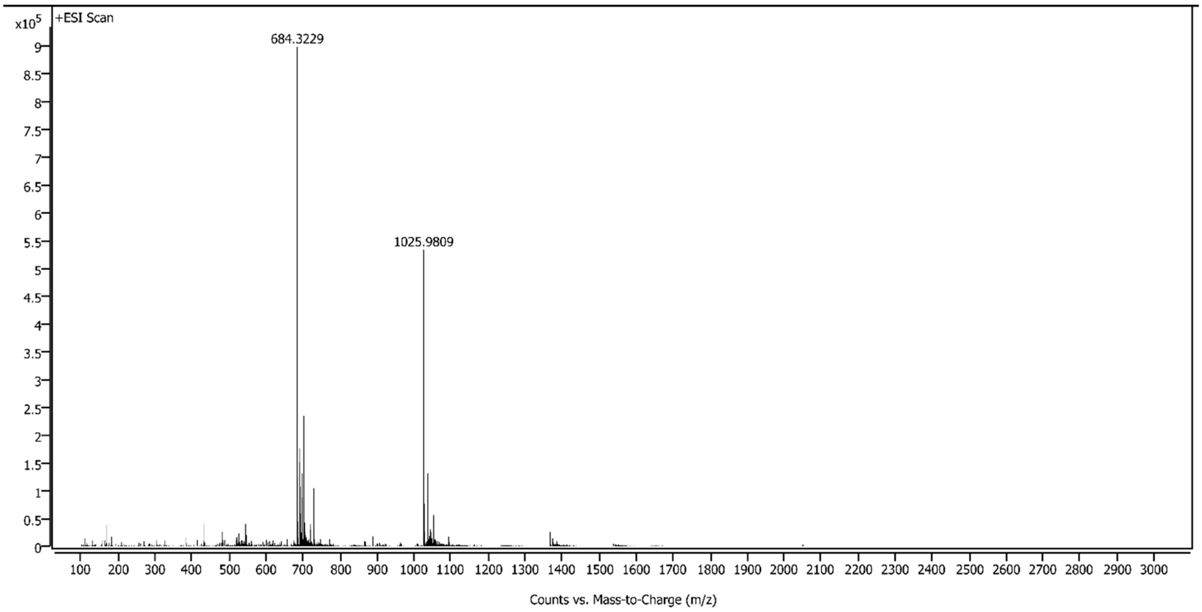

Spectrum Peaks

m/z	Z	Abund	Abund %	m/z (Calc)	Diff (ppm)	Ion Species	Formula
683.9885	3	823526	91.56				
684.3229	3	899475	100.00	684.3233	0.58	$[M+3H]^{3+}$	$C_{90}H_{136}N_{24}O_{27}S_2$
684.6568	3	600665	66.78				
684.9905	3	306066	34.03				
1025.4794	2	494067	54.93				
1025.9809	2	535168	59.50				
1026.4817	2	355016	39.47				
1026.9824	2	181881	20.22				

Figure S21: HRMS spectrum of **SulfoCy3-TJ12P2(Q[11])**.

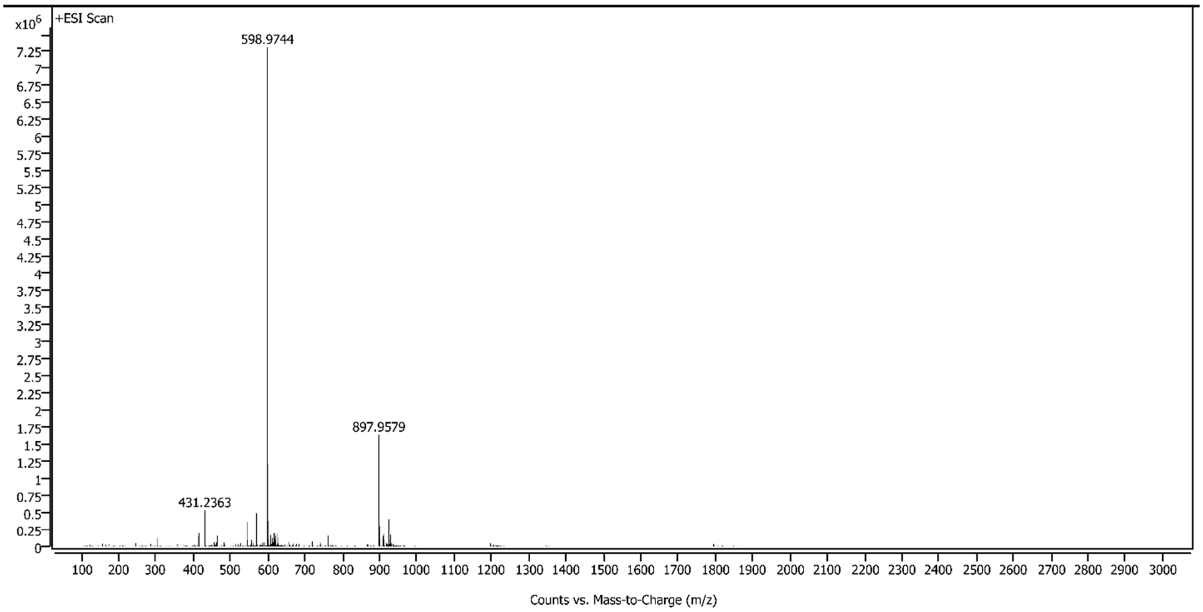

Spectrum Peaks

m/z	Z	Abund	Abund %	m/z (Calc)	Diff (ppm)	Ion Species	Formula
598.9744	3	7316344	100.00	598.9746	0.33	$[M+3H]^{3+}$	$C_{74}H_{123}N_{25}O_{27}$
599.3086	3	6740160	92.12				
599.6427	3	3334534	45.58				
599.9768	3	1213930	16.59				
897.9579	2	1640528	22.42				
898.4591	2	1508242	20.61				
898.9603	2	784727	10.73				

Figure S22: HRMS spectrum of **NODA-GA-TJ12P2(Q[11])**.

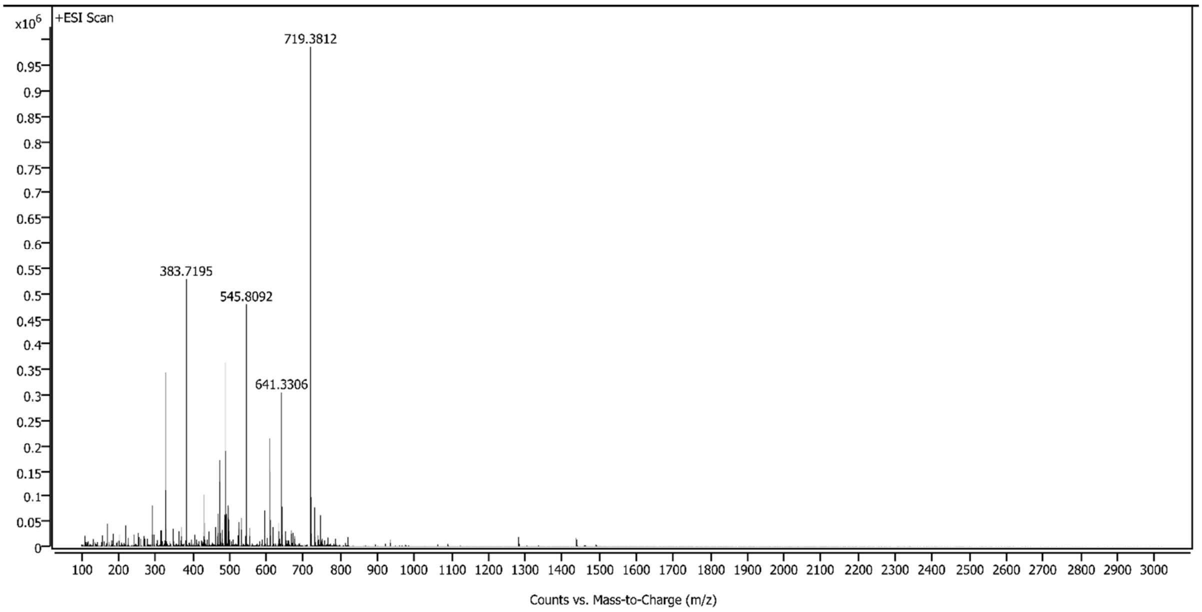

Spectrum Peaks

m/z	Z	Abund	Abund %	m/z (Calc)	Diff (ppm)	Ion Species	Formula	Ion Type
719.3812	2	987424	100.00	719.3815	0.42	$[M+2H]^{2+}$	$C_{59}H_{100}N_{22}O_{20}$	
719.8826	2	722193	73.14					
720.3838	2	307773	31.17					
720.8852	2	97447	9.87					

Figure S23: HRMS spectrum of ***scr*-TJ12P2(Q[11])**.

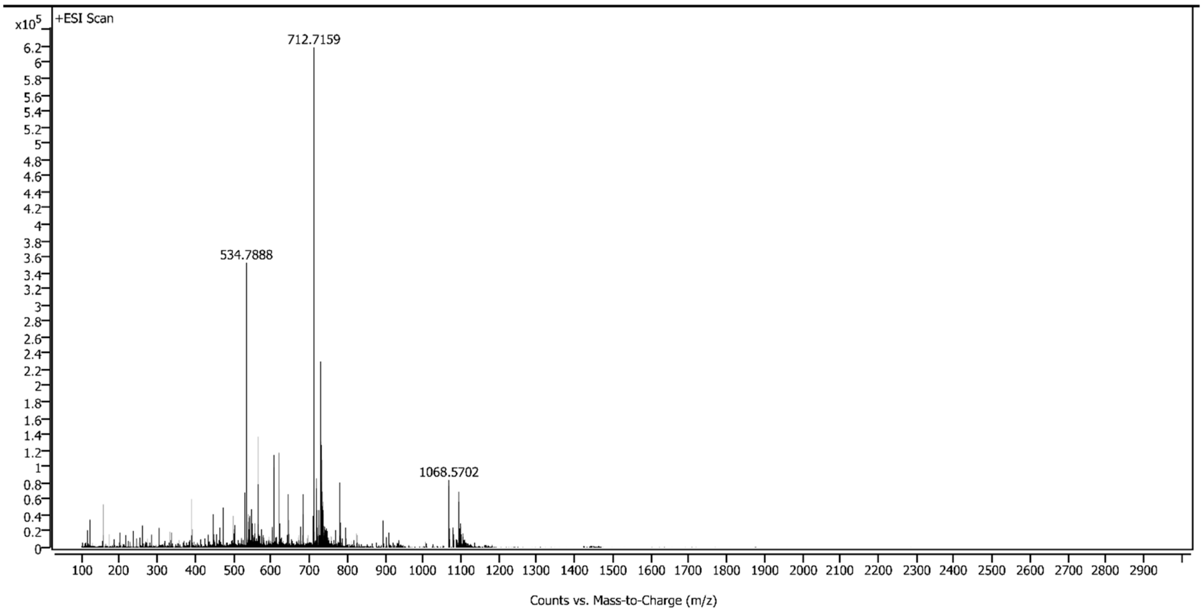

Spectrum Peaks

m/z	Z	Abund	Abund %	m/z (Calc)	Diff (ppm)	Ion Species	Formula
712.3817	3	559679	90.46				
712.7159	3	618721	100.00	712.7165	0.84	$[M+3H]^{3+}$	$C_{89}H_{151}N_{31}O_{30}$
713.0502	3	383869	62.04				
713.3840	3	175876	28.43				
534.5381	4	320707	51.83				
534.7888	4	352639	56.99				
535.0394	4	226416	36.59				
535.2899	4	102887	16.63				
1068.0689	2	75780	12.25				
1068.5702	2	83620	13.52				

Figure S24: HRMS spectrum of **TJ12P2(Q[11])-X_3K(NODA-GA)-$CONH_2$**.

Table S1: Synthesized peptide constructs with corresponding sequences and chemical formulas, calculated and found high resolution masses (m/z 100%) and chemical purity as determined by analytical HPLC (220 nm).

Peptide	Sequence	Chemical Formula	m/z calc.	m/z found	Purity [%]
**TJ12P2(K^{11})**	SNDRPPNILQKR-OH	$C_{60}H_{104}N_{22}O_{19}$	719.3997 $[M+2H]^{2+}$	719.3994	≥99.8
**SulfoCy3-TJ12P2(K^{11})**	SulfoCy3-SNDRPPNILQKR-OH	$C_{91}H_{140}N_{24}O_{26}S_2$	684.3355 $[M+3H]^{3+}$	684.3349	≥98.2
**NODA-GA-TJ12P2(K^{11})**	NODA-GA-SNDRPPNILQKR-OH	$C_{75}H_{127}N_{25}O_{26}$	598.9868 $[M+3H]^{3+}$	598.9865	≥98.5
***Scr*-TJ12P2(K^{11})**	DRNSQKINPRPL-OH	$C_{60}H_{104}N_{22}O_{19}$	479.9356 $[M+3H]^{3+}$	479.9354	≥99.0
**TJ12P2(K^{11})-X$_3$K(NODA-GA)-CONH$_2$**	SNDRPPNILQKRXXXK(NODA-GA)-CONH$_2$	$C_{90}H_{155}N_{31}O_{29}$	712.3942 $[M+3H]^{3+}$	712.3936	≥98.6
**TJ12P2(Q^{11})**	SNDRPPNILQQR-OH	$C_{59}H_{100}N_{22}O_{20}$	719.3815 $[M+2H]^{2+}$	719.3813	≥99.1
**SulfoCy3-TJ12P2(Q^{11})**	SulfoCy3-SNDRPPNILQQR-OH	$C_{90}H_{136}N_{24}O_{27}S_2$	684.3233 $[M+3H]^{3+}$	684.3229	≥99.8
**NODA-GA-TJ12P2(Q^{11})**	NODA-GA-SNDRPPNILQQR-OH	$C_{74}H_{123}N_{25}O_{27}$	598.9746 $[M+3H]^{3+}$	598.9744	≥96.4
***Scr*-TJ12P2(Q^{11})**	RRPQDLPINQNS-OH	$C_{59}H_{100}N_{22}O_{20}$	719.3815 $[M+2H]^{2+}$	719.3812	≥99.0
**TJ12P2(Q^{11})-X$_3$K(NODA-GA)-CONH$_2$**	SNDRPPNILQQRXXXK(NODA-GA)-CONH$_2$	$C_{89}H_{151}N_{31}O_{30}$	712.7165 $[M+3H]^{3+}$	712.7159	≥98.5

S2 Nanobody synthesis

Nanobody design/expression

Cloning of the anti-GPC3 nanobody HN3 into the bacterial expression vector pET-28b, their cytoplasmic expression in *Escherichia* (*E.*) *coli* SHuffle T7 Express as well as their purification by affinity chromatography were described in detail previously [1]. The coding sequence for HN3 was purchased from Eurofins Genomics with 5′ *Nco*I and 3′ *Hin*dIII restriction enzyme sites for cloning into the vector pET-28b:Sortag-TwinStrep. This plasmid was derived from the commercially available pET-28b vector (Merck) by addition of a DNA fragment coding for a $(GGGGS)_3$ spacer followed by the LPETGG sortase motif, another $(GGGGS)_3$ spacer and the Twin-Strep-tag WSHPQFEK-$(GGGS)_2$-GGSA-WSHPQFEK. Following ligation into pET-28b:Sortag-TwinStrep, plasmid DNA was transformed into *E. coli* NEB 5-alpha cells (New England Biolabs) and the sequence of the recombinant constructs was verified by DNA sequencing (Eurofins Genomics). For cytoplasmic expression of proteins, the vectors were transformed into *E. coli* SHuffle T7 Express (New England Biolabs) and MagicMedia E. coli Expression Medium (Life Technologies) was used according to the manufacturer's protocol. After expression, the recombinant nanobody **HN3-sortag-(Strep)$_2$** was purified by affinity chromatography using a high-capacity Strep-Tactin®XT resin (IBA Lifesciences GmbH) in combination with an ÄKTA pure chromatography system (GE Healthcare).

Site-specific functionalization of nanobodies

The two-step site-specific antibody modification approach using a combination of Sortase A-mediated bioconjugation and strain-promoted azide-alkyne cycloaddition (SPAAC) was performed as recently described with a few modifications [1]. Briefly, an azidolysine-containing triglycine peptide (10.0 eq, Iris Biotech GmbH), HIS-tagged sortase A Δ59 (1.00 eq) and **HN3-sortag-(Strep)$_2$** (1.00 eq) were incubated at 30 °C for 4 h in sortase buffer (50 mM Tris-HCl, 150 mM NaCl, 10 mM $CaCl_2$, pH 7.5). Subsequently, the HIS-tagged enzyme was removed from the reaction mixture by immobilized-metal affinity chromatography (IMAC) using His60 Nickel gravity columns (Clontech Laboratories). Unreacted excess peptide was removed by spin filtration using centrifugal filter devices with a molecular weight cut-off of 3 kDa (Amicon Ultra 3 kDa cutoff, Merck). The resulting azide-functionalized nanobodies were reacted either with DBCO-SulfoCy3 (10.0 eq, Jena Bioscience GmbH) or BCN-NODA-GA (10.0 eq, Chematech) in sortase buffer at 25 °C for 4 h. Excess cycloaddition reagents were separated from the desired nanobody construct by size-exclusion chromatography using Zeba Spin Desalting Columns (7 K, Thermo Scientific) with elution in PBS (**Sulfo-Cy3-HN3**) or 0.2 M ammonium acetate buffer (pH 6, **NODA-GA-HN3**) and subsequent spin filtration as described above.

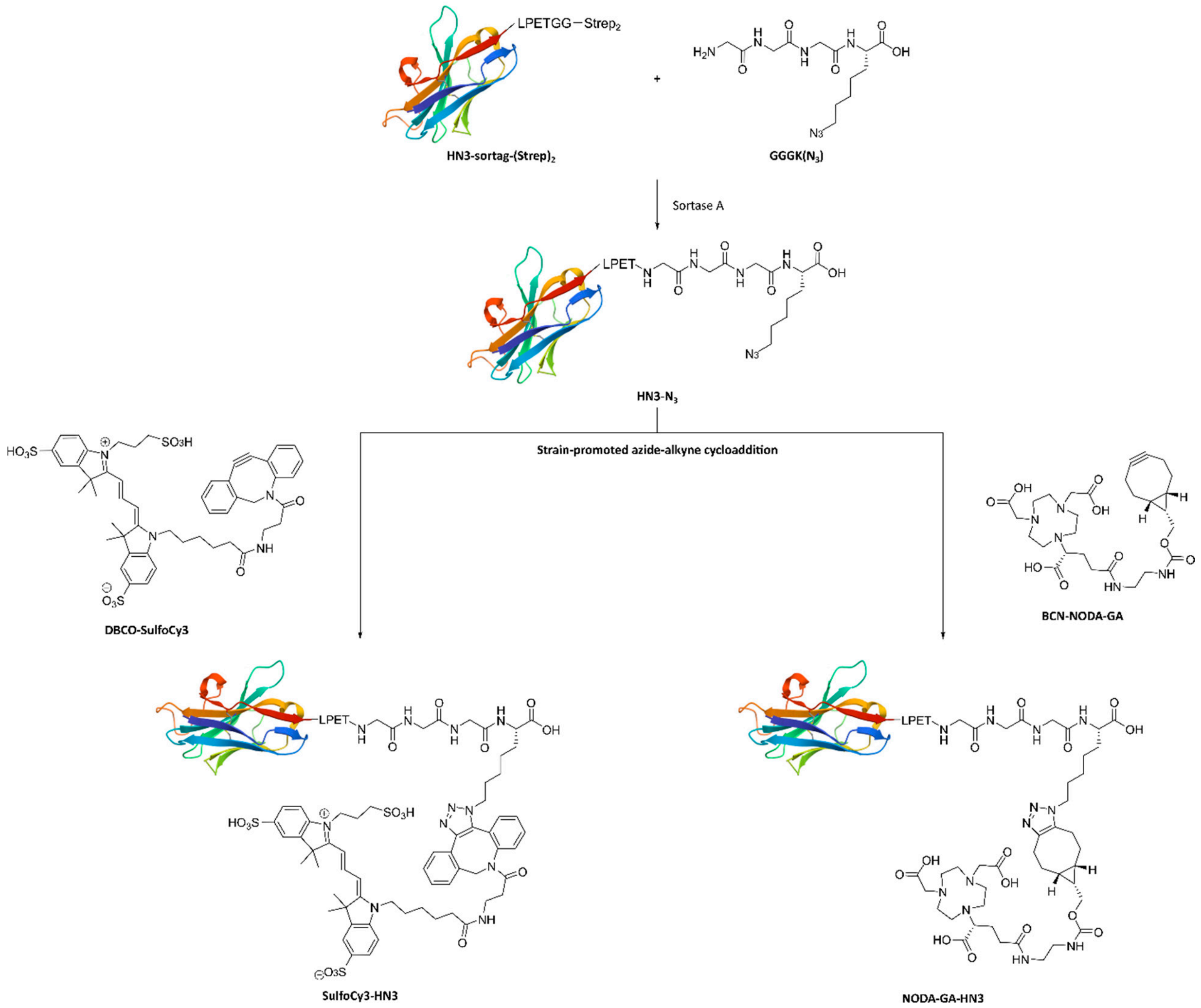

Figure S25: Site-specific modification of **HN3-sortag-(Strep)2** carrying a C-terminal LPETG sortase motif and a Twin-Strep-tag (Strep)2 by Sortase A-mediated bioconjugation and subsequent strain-promoted azide-alkyne cycloaddition.

S3 additional Information

Single cycle kinetics in SPR

Binding was analyzed using the same chip as described in the main manuscript by capturing His-tagged human GPC3 (GP3-H52H4, Acro Biosystems) as ligand on the active FC and subsequently conducting a single-cycle kinetic on both FC. For determination of nanobody dissociation constants, three start-up cycles were performed at the beginning of each experiment. Each cycle consisted of capturing His-tagged hGPC3 (5 µg/mL in HBS-P+, 30-60 s; 10 µL/min) on FC(active) followed by nanobody association (60 s) on both FC, a regeneration period (10 mM glycine, pH = 1.5, 30 s) and stabilization of 300 s. Binding analysis cycles were carried out starting with hGPC3 (5 µg/mL in HBS-P+, 30-60 s; 10 µL/min) on FC(active). Then, five increasing concentrations of the respective nanobody were injected in both flow cells in a kinetic titration, where both FC were exposed to each concentration for 90 s followed by a dissociation phase of 300 s. Employed concentrations were in the range of 0.24 – 150 nM (5-fold dilution series), and analyses were carried out in triplicates for two independent experiments. Regeneration of the chip was carried out as described above. For double referencing, blank runs using only buffer (HBS-P+) were performed.

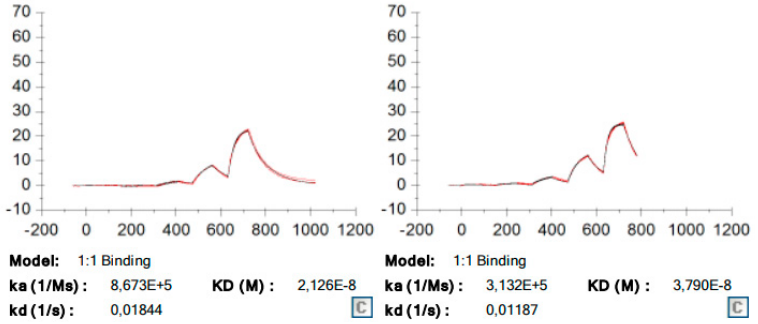

Figure S26: Double-referenced sensorgrams of single cycle kinetics of **HN3-sortag-(Strep)$_2$** at different concentrations 0.24 – 150 nM) with kinetic parameters obtained by nonlinear regression. Horizontal axis is time [s], vertical axis is absolute response unit [RU].

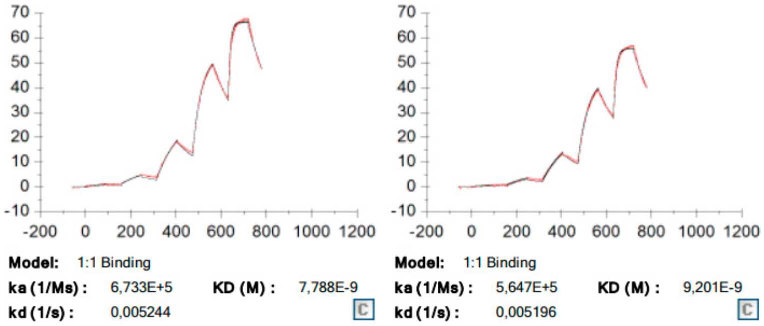

Figure S27: Double-referenced sensorgrams of single cycle kinetics of **NODA-GA-HN3** at different concentrations 0.24 – 150 nM) with kinetic parameters obtained by nonlinear regression. Horizontal axis is time [s], vertical axis is absolute response unit [RU].

Table S2: Association and dissociation rate constants as well as calculated equilibrium dissociation constant for HN3 versions as determined by single-cycle kinetic of different concentrations (0.24 – 150 nM) in SPR.

	k_{on} [$M^{-1}s^{-1}$]	k_{off} [s^{-1}]	K_D [nM]
HN3-sortag-(Strep)$_2$	5.90 x10^5	1.52 x10^{-2}	29.6
NODA-GA-HN3	6.19 x10^5	5.22 x10^{-3}	8.50

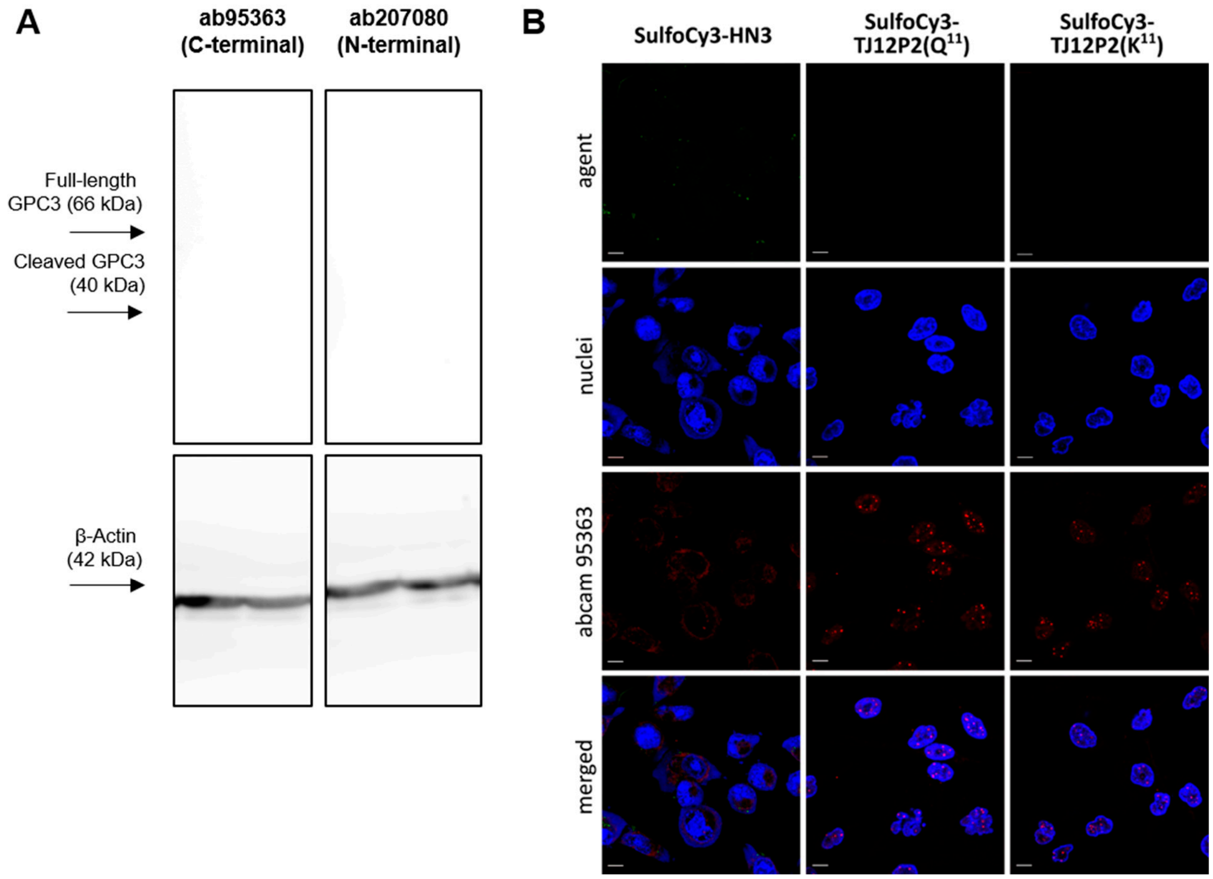

Figure S28: (**A**) Western Blot images generated using primary ies binding C-terminal (ab95363) or N-terminal (ab207080) epitopes of GPC3 in two different Sk-Hep1 lysates. β-Actin was used as loading control. Uncropped/Whole Western Blot images are provided in **Figure S29** and **Figure S30:** . (**B**) Fluorescence microscopy images of GPC3-positive HepG2 cells stained using **SulfoCy-3-HN3**, **SulfoCy3-TJ12P2(Q^{11})** and **SulfoCy3-TJ12P2(K^{11})** (green) after 2 h of incubation. Nuclei are depicted in blue, control stain of GPC3 using ab95363 is depicted in red.

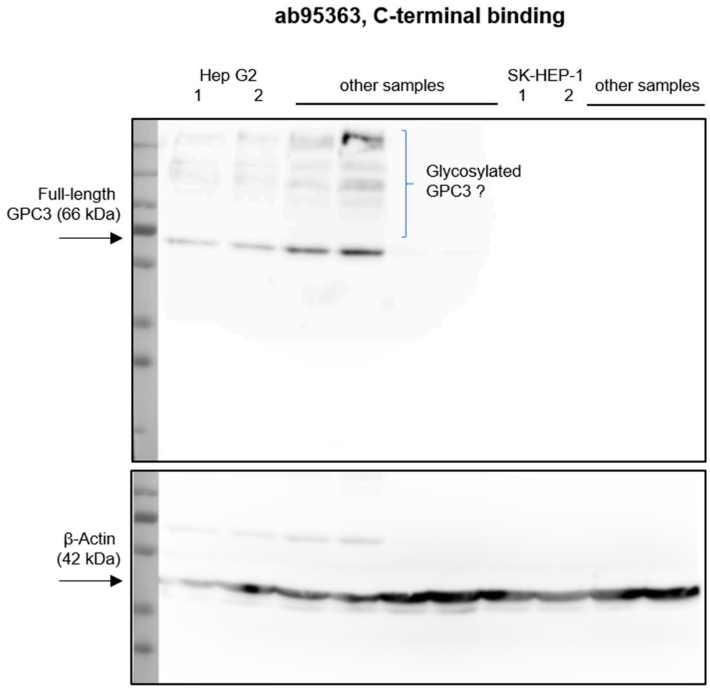

Figure S29: Uncropped original Western blot images for ab95363 containing lanes depicted in **Figure 3** and **Figure S28** and additional lanes not discussed in this publication.

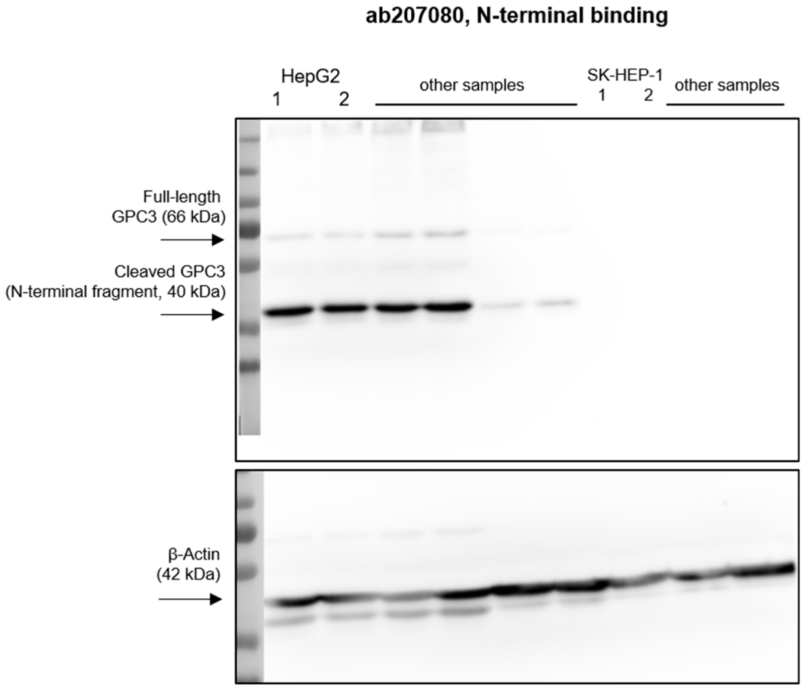

Figure S30: Uncropped original Western blot images for ab207080 containing lanes depicted in **Figure 3** and **Figure S28** and additional lanes not discussed in this publication.

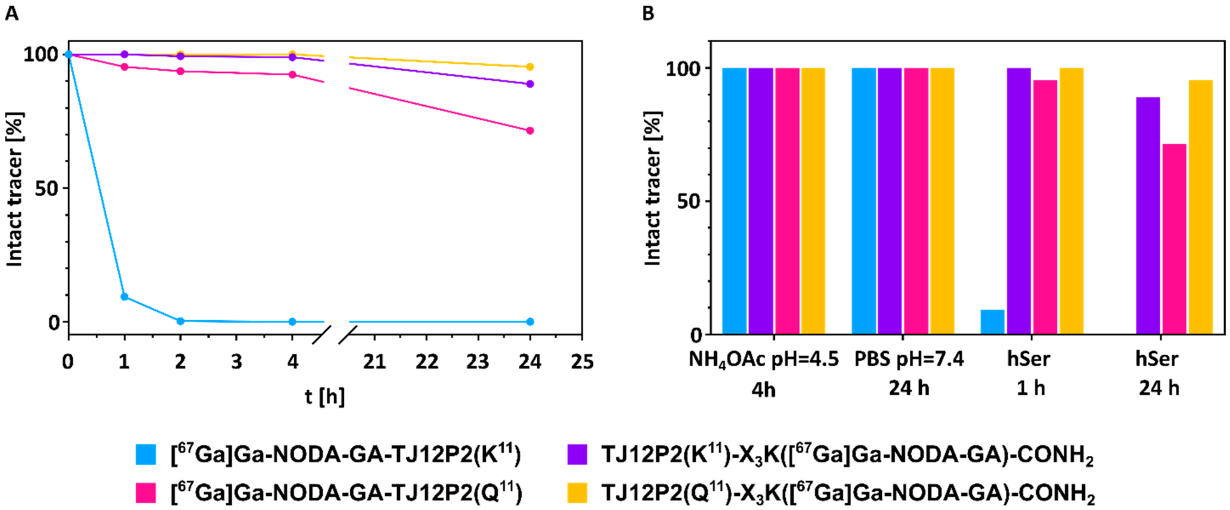

Figure S31: (A) Stability of radiolabeled peptide in human serum at 37 °C over 24 h. **(B)** Stability of radiolabeled peptides in NH_4OAc buffer, PBS and human serum.

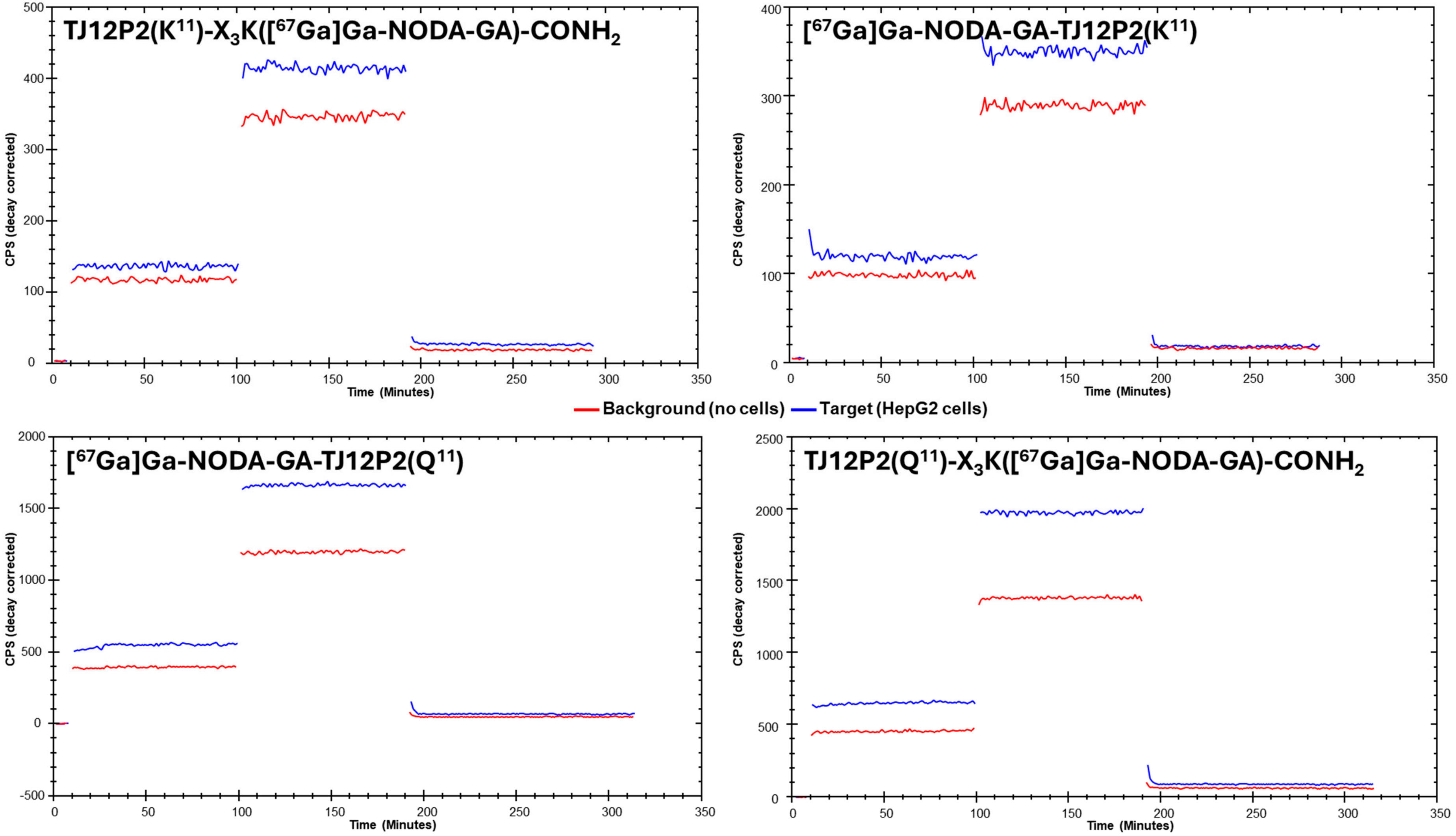

Figure S32: Exemplary raw data (CPS) of real-time radioligand binding experiments of all four compounds. Applied concentrations were 250 nM and 750 nM in the first and second association phase, respectively. Data shows no differences in pattern between Target (HepG2 cells) and Background (no cells) area, especially no signal increase over time, therefore indicating a lack of specific binding.

1. Singh, G., Zarschler, K., Hunoldt, S., Martínez, I. I. S., Ruehl, C. L., Matterna, M., Bergmann, R., Máthé, D., Hegedüs, N., Bachmann, M., et al. Versatile Bispidine-Based Bifunctional Chelators for 64CuII-Labelling of Biomolecules. *Chemistry – A European Journal* **2020**, *26*, 1989-2001, doi:https://doi.org/10.1002/chem.201904654.
